# Early adversity promotes binge-like eating habits by remodeling a leptin-responsive lateral hypothalamus–brainstem pathway

**DOI:** 10.1038/s41593-022-01208-0

**Published:** 2022-12-12

**Authors:** Sora Shin, In-Jee You, Minju Jeong, Yeeun Bae, Xiao-Yun Wang, Mikel Leann Cawley, Abraham Han, Byung Kook Lim

**Affiliations:** 1 Fralin Biomedical Research Institute at VTC, Roanoke, VA USA; 2 FBRI Center for Neurobiology Research, Roanoke, VA USA; 3 Department of Human Nutrition, Foods, and Exercise, Virginia Polytechnic Institute and State University, Blacksburg, VA USA; 4 Neurobiology Section, Division of Biological Sciences, University of California, San Diego, La Jolla, CA USA

**Keywords:** Hypothalamus, Stress and resilience

## Abstract

Early-life trauma (ELT) is a risk factor for binge eating and obesity later in life, yet the neural circuits that underlie this association have not been addressed. Here, we show in mice that downregulation of the leptin receptor (Lepr) in the lateral hypothalamus (LH) and its effect on neural activity is crucial in causing ELT-induced binge-like eating and obesity upon high-fat diet exposure. We also found that the increased activity of Lepr-expressing LH (LH^Lepr^) neurons encodes sustained binge-like eating in ELT mice. Inhibition of LH^Lepr^ neurons projecting to the ventrolateral periaqueductal gray normalizes these behavioral features of ELT mice. Furthermore, activation of proenkephalin-expressing ventrolateral periaqueductal gray neurons, which receive inhibitory inputs from LH^Lepr^ neurons, rescues ELT-induced maladaptive eating habits. Our results identify a circuit pathway that mediates ELT-induced maladaptive eating and may lead to the identification of novel therapeutic targets for binge eating and obesity.

## Abstract (web summary)

Early-life trauma is a risk factor for binge eating and obesity later in life. Shin et al. identify a hypothalamus–brainstem circuit in mice that underlies the early-life trauma-induced binge-like consumption of high-fat diet and obesity-prone characteristics.

## Main

Stress has a powerful effect on food consumption, dietary preferences and food responsiveness^1^. In particular, exposure to traumatic events early in life, including child maltreatment or physical abuse, is a major risk factor for the development of maladaptive eating habits like emotional overeating and binge eating in adulthood^2–4^.

Binge eating is characterized by the emotionally triggered consumption of large amounts of foods in a short period. Binge eating disorder (BED), the most common type of eating disorder, is defined by recurrent episodes of binge eating accompanied by feelings of loss of control even in the absence of hunger^5^, and it is often associated with being overweight^6^. Importantly, clinical studies suggest adults with BED frequently report histories of certain forms of ELT (for example, family conflict, loss of family members or economic distress), highlighting the importance of ELT in the development of binge eating habits later in life^7,8^. Thus, identifying the specific neural mechanisms underlying ELT-induced binge eating may aid the development of more effective therapies for BED and obesity.

Because ELT is associated with long-lasting changes in the activity of the hypothalamic–pituitary–adrenal (HPA) axis, which influences the hormones that regulate appetite such as leptin^9,10^, studies have examined the mechanisms by which ELT alters the feeding-related hormonal system to induce maladaptive eating behaviors^11,12^. Leptin acts as a trophic factor during early development^13^, and a surge in circulating leptin levels regulates gene expression and synaptic connectivity crucial for the development of hypothalamic circuits^14^. Given that the hypothalamus is central in the regulation of feeding behaviors^15^, it is plausible that ELT may disturb the leptin system and impair hypothalamic function, with long-term consequences for appetite control and weight gain. However, leptin-responsive neural mechanisms or hypothalamic pathways that may drive ELT-induced binge eating habits have not been explored.

Leptin exerts its anorexic effects by acting on its receptors (Lepr) in the hypothalamus. Lepr activation stimulates multiple signal transduction pathways and regulates neuronal activity to suppress food intake and body weight gain^16,17^. The arcuate nucleus (Arc) is one of the hypothalamic subregions in which considerable effort has been made to elucidate the central role of Lepr^18^; however, the functions of Lepr beyond the Arc in the pathophysiology of BED are unknown. Indeed, Lepr is widely expressed in several hypothalamic subregions, including the LH, dorsomedial hypothalamus (DMH) and ventromedial hypothalamus (VMH)^19^. This wide hypothalamic distribution indicates that Lepr signaling may be involved in various functions, including regulation of stress responses, reward processing and emotional states^20^. However, apart from the homeostatic control of feeding, how leptin-responsive hypothalamic pathways may underlie emotional overeating and adapt to ELT has not been addressed.

The LH is a heterogeneous structure that contains genetically and functionally distinct cell populations^21,22^. Early work with electrolytic lesions and electrical stimulation identified the LH as a crucial area for regulating food intake and motivated behaviors^23^. The LH also modulates the HPA axis to coordinate behavioral and physiological responses to stressful events^24^. However, the specific cell types or the precise LH circuit mechanisms that transduce ELT into a maladaptive circuit state that leads to binge eating and obesity later in life have not been explored.

Here, we show in mice that ELT impairs Lepr signaling in the LH and that impaired LH^Lepr^ signaling is sufficient to replicate the binge-like eating habits and accelerated weight gain of ELT mice. Moreover, LH^Lepr^ neurons of ELT mice show consistent activation in response to repetitive high-fat diet (HFD) reexposures, which was associated with sustained binge-like eating patterns. In addition, acute and chronic silencing of the ventrolateral periaqueductal gray (vlPAG)-projecting LH^Lepr^ neurons attenuates the maladaptive eating habits and excessive body weight gain in ELT mice. Finally, we show that vlPAG neurons expressing the opioid peptide proenkephalin (Penk) are important downstream targets of LH^Lepr^ neurons that mediate the effect of ELT on binge-like eating habits. Together, our findings identify a discrete leptin-responsive LH–vlPAG pathway that is an important mediator of ELT-induced binge-like HFD overconsumption and weight gain.

## Results

### Early-life trauma augments binge-like eating in adulthood

**Fig. 1:**
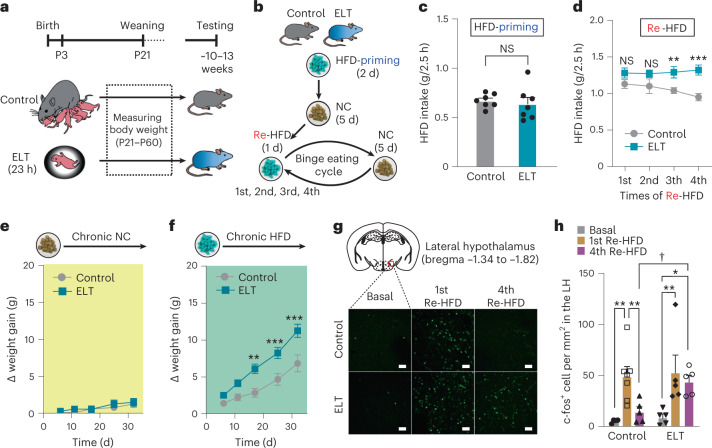
ELT enhanced binge-like eating and weight gain, accompanied by increased c-fos in the LH after repetitive cycles of Re-HFD. **a**, The experimental timeline of the ELT procedure. ELT pups were removed and separated for 23 h both from their dam and littermates at P3. **b**, Diagram of repetitive cycles of Re-HFD to induce binge-like eating. After 2 d of HFD priming, the HFD was removed, but NC was available. Mice received four successive binge eating cycles by access to Re-HFD for 1 d after 5 d of ad libitum NC only. **c**,**d**, 2.5 h HFD consumption in response to either the first exposure to HFD (**c**) or Re-HFD (**d**; *n* = 7 mice per group). Two-way repeated-measures (RM) analysis of variance (ANOVA; in **d**, *F*_(1,36) _= 22.385, *P* < 0.001) was followed by Bonferroni post hoc test for multiple comparisons; **P = 0.01, ***P < 0.001, compared with control mice at respective Re-HFD cycles. **e**,**f**, Cumulative body weight gain of control versus ELT mice given ad libitum access to either NC (**e**; *n* = 8, 11 mice per group) or HFD (**f**; *n* = 10, 12 mice per group). Two-way RM ANOVA (in **f**, *F*_(1,80) _= 12.647, *P* = 0.002) was followed by Bonferroni post hoc test for multiple comparisons; **P = 0.002, ***P < 0.001, compared with control mice at the respective days. **g**, Example images of the LH showing c-fos staining at the basal state or following Re-HFD. Scale bars, 50 μm. **h**, Quantification of c-fos-positive cells in the LH (*n* = 4, 5 mice for each basal group, *n* = 7, 5 mice for each 1st Re-HFD group and *n* = 5, 5 mice for each 4th Re-HFD group). Two-way ANOVA (*F*_(2,25) _= 11.109, *P* < 0.001) was followed by Fisher’s least significant difference (LSD) post hoc test for multiple comparisons; **P = 0.003, **P = 0.009, compared with control mice at the 1st Re-HFD; **P = 0.003, *P = 0.015, compared with ELT mice at basal condition; ^†^*P* = 0.036 compared with control mice at the 4th Re-HFD. NS, not significant. Data are the mean ± s.e.m.

**Extended Data Fig. 1:**
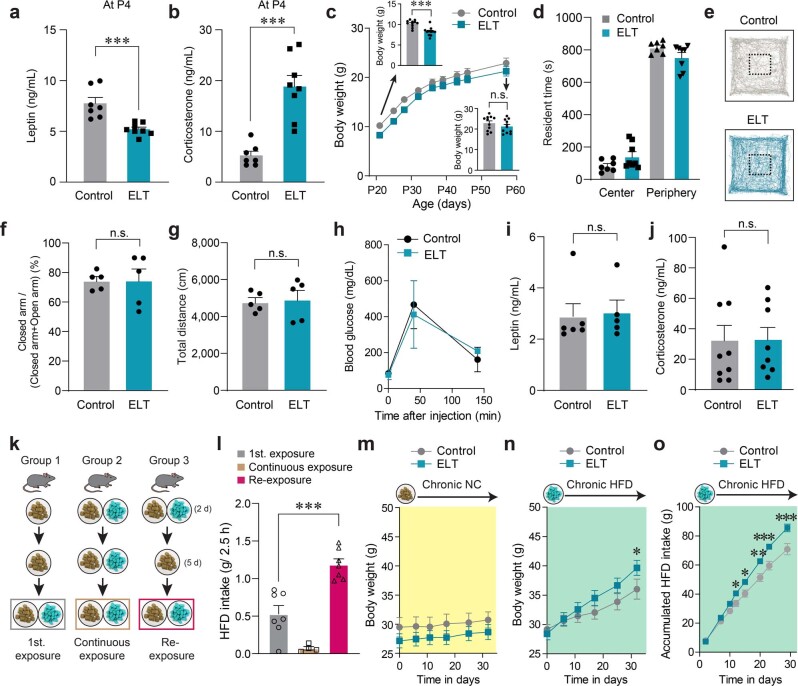
Basic characteristics of ELT mice. (**a, b**) Serum leptin (**a**) and corticosterone (**b**) level of mouse pups at P4 (n = 7, 8 mice per group). Two-tailed unpaired t-test; in a, t13 = 4.413, ***p = 0.000701; in b, t13 = −5.502, ***p = 0.000102. (**c**) Body weight changes of control and ELT mice fed NC from weaning (21 days old) to adulthood (8 weeks old) (n = 11 mice per group). Two-tailed unpaired t-test, t20 = 4.953, ***p = 0.0000765. (**d, e**) The resident time spent in the center or periphery area of the open field (d; n = 7, 8 mice per group) and representative movement tracks from control and ELT mice (**e**). Two-way RM ANOVA (F(1,13)= 0.116, p = 0.739); p = 0.09 for control versus ELT in center; p = 0.09 for control versus ELT in periphery zone. (**f**) Percentage time spent in the closed arms of the elevated plus maze (n = 5 mice per group). Two-tailed unpaired t-test, t8 = −0.0198, p = 0.985. (**g**) Locomotor activity is monitored and total distance traveled is measured during the 15 min test period (n = 5 mice per group). Two-tailed unpaired t-test, t8 = −0.250, p = 0.809. (**h**) Glucose tolerance levels between controls and ELT mice (n = 3 mice per group). (**i, j**) Adult ELT mice show the normal levels of serum leptin (**i**) and corticosterone (**j**) (n = 9, 8 mice per group). Two-tailed unpaired t-test; in i, t15 = −0.416, p = 0.683; in j, t15 = −0.0440, p = 0.965. (**k**) Diagram of the diet schedule to induce binge-like eating in mice. Mice in Group 3 are subjected to an intermittent HFD access schedule. On day 8, the HFD is presented to mice in all groups. (**l**) HFD intake in 2.5 h after the presentation of the HFD to mice in Group 1, 2 and 3 (**k**) on day 8 (n = 7, 4 and 7 mice per group). One-way ANOVA (F(2,15)= 32.824, p < 0.001) was followed by Fisher LSD post hoc test for multiple comparisons; ***p < 0.001 compared with the 1st HFD exposure. (**m, n**) Body weight changes of adult control and ELT mice fed NC (m; n = 8, 11 mice per group) or HFD (n; n = 10, 12 mice per group). Two-way RM ANOVA (in n, F(5,100)= 6.855, p < 0.001) was followed by Bonferroni post hoc test for multiple comparisons; *p = 0.043 compared with control at the respective day. (**o**) Cumulative HFD intake under chronic HFD (n = 6, 5 mice per group). Two-way RM ANOVA (F(7,63)= 11.704, p < 0.001) was followed by Bonferroni post hoc test for multiple comparisons; *p = 0.041, *p = 0.020, **p = 0.002, ***p < 0.001 compared with controls at respective days. n.s., not significant. Data are presented as mean ± SEM.

To investigate the effects of ELT on binge eating habits in adults, we adopted a modified version of the ELT paradigm in which mouse pups at postnatal day 3 (P3) were subjected to 23 h of separation from their dam and littermates, thus exposing pups to conditions with deficits in emotional and physical care^25^ (Methods and Fig. 1a). To examine the impact of ELT on the modulation of peripheral hormones associated with feeding and stress, we measured serum leptin and corticosterone levels at the end of the ELT session. ELT pups had lower leptin and higher corticosterone levels than control pups at P4 (Extended Data Fig. 1a,b). Altered levels of these hormones in early life are known to have long-lasting effects on body weight or behavioral stress responses^26,27^. The ELT mice also showed reduced body weight at weaning (P21), but this difference disappeared such that by ~8 weeks old ELT mice and controls had similar body weights (Extended Data Fig. 1c). In addition, adult ELT mice did not show differences in anxiety-like behaviors, locomotion, glucose metabolism and baseline leptin and corticosterone levels compared with controls (Extended Data Fig. 1d–j).

The consumption of palatable foods can contribute to emotional overeating in humans and animals^28,29^. Scheduled intermittent access to HFD (60% kcal% fat) induced rapid binge-like eating behaviors in mice, consistent with previous studies^30^. That is, upon reexposure to HFD (Re-HFD), adult mice from the intermittent-access group quickly consumed larger amounts of HFD in 2.5 h than mice exposed to a HFD for the first time or to a HFD continuously (Extended Data Fig. 1k,l). This suggests that mice show a greater tendency toward binge-like eating under HFD exposure, particularly when palatable foods are presented back to them. We next tested whether adult ELT mice (~10–13 weeks old) show exacerbated binge-like HFD consumption. Given that BED is characterized by recurrent binge eating episodes^5^, we applied multiple cycles of intermittent HFD access to determine whether ELT induces repetitive binge-like eating habits (Fig. 1b). For the first-time exposure to HFD (HFD priming), ELT and control mice showed similar HFD intake (g/2.5 h; Fig. 1c). However, in response to Re-HFD, ELT mice showed aggravated binge eating tendencies over repetitive cycles, which was particularly manifested in the 4th cycle of Re-HFD, but not in the 1st cycle (Fig. 1d). This suggests that ELT mice show maladaptive eating habits with augmented and sustained binge-like eating patterns.

**Extended Data Fig. 2:**
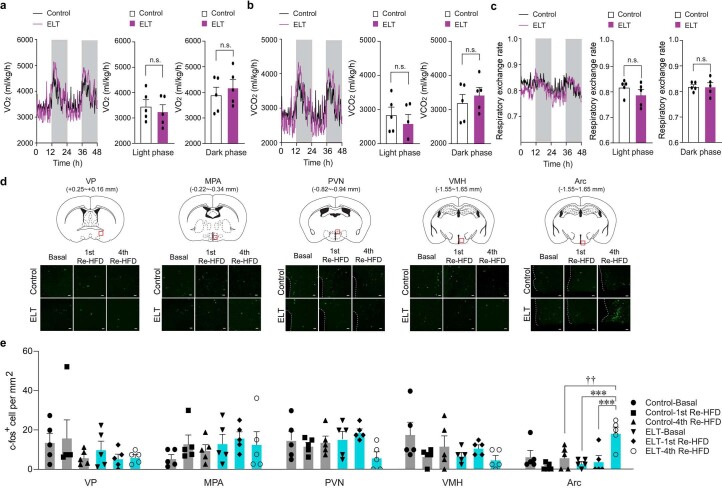
Energy expenditure in ELT mice and quantification of c-fos-positive cells after repetitive cycles of intermittent HFD access. (**a-c**) Oxygen consumption (VO2; a), carbon dioxide production (VCO2; b) and respiratory exchange rate (RER; c) measurements in control and ELT mice given HFD and kept in metabolic cages for 2 dark and light cycles (n = 5 mice per group). Two-tailed unpaired t-test, in a, light phase, t8 = 0.550, p = 0.598; in a, dark phase, t8 = −0.678, p = 0.517; in b, light phase, t8 = 0.707, p = 0.499; in b, dark phase, t8 = −0.717, p = 0.494; in c, light phase, t8 = 1.192, p = 0.267; in c, dark phase, t8 = −0.0197, p = 0.985. (**d**) Coronal diagrams depicting the brain region analyzed for quantifying c-fos-positive cells (red square; top). Example images of the VP, MPA, PVN, VMH and Arc showing c-fos staining in control versus ELT mice at the basal state or following the 1st and 4th cycles of Re-HFD. Scale bars, 25 μm. (**e**) Quantifications of c-fos-positive cells in the VP, MPA, PVN, VMH and Arc between control and ELT mice (n = 5 mice per group). Two-way ANOVA (F(2,24)= 5.519, p = 0.011) was followed by Fisher LSD post hoc test for multiple comparisons; in the Arc, ***p < 0.001 compared with ELT mice at the 4th Re-HFD; ††p = 0.001 compared with control mice at the 4th Re-HFD. n.s., not significant. Data are presented as mean ± SEM.

Binge eating habits are associated with being overweight^6^. Consistent with this, male and female ELT mice showed rapid weight gain when fed a HFD, but not under chronic access to normal chow (NC; Fig. 1e,f and Extended Data Fig. 1m,n). In addition, ELT mice consumed more HFD without any concurrent differences in energy expenditure, such as oxygen consumption, carbon dioxide production and respiratory exchange rate (Extended Data Fig. 1o and 2a–c). These data strongly suggest that ELT mice are susceptible to the development of HFD-induced obesity, primarily due to overconsumption of the foods.

### Roles of Lepr in the lateral hypothalamus on binge-like eating and obesity

Animal studies and human neuroimaging studies indicate that the consumption of palatable foods can activate neural activity in several brain areas^31,32^. We therefore hypothesized that ELT alters neural activity in specific brain areas to increase HFD responsiveness. To test this, we first examined the expression of the neuronal activation marker c-fos in several brain areas involved in feeding behaviors^33–36^ after Re-HFD in repetitive cycles of intermittent HFD access. In the LH, ELT mice showed stably elevated c-fos expression in both the 1st and 4th Re-HFD, whereas controls exhibited increased c-fos after 1st Re-HFD, but only mild enhancements at the 4th Re-HFD (Fig. 1g,h). Other brain areas, such as the ventral pallidum, medial preoptic area (MPA), paraventricular nucleus and VMH, show ambiguous differences in c-fos patterns across the Re-HFD cycles, while the Arc of ELT mice displayed elevated c-fos expression in the 4th Re-HFD cycle (Extended Data Fig. 2d,e). Given our previous data showing augmented and sustained binge-like HFD consumption in ELT mice with multiple Re-HFD cycles, these c-fos data indicate that the LH or Arc activation is potentially involved in ELT-induced binge-like eating habits.

**Extended Data Fig. 3:**
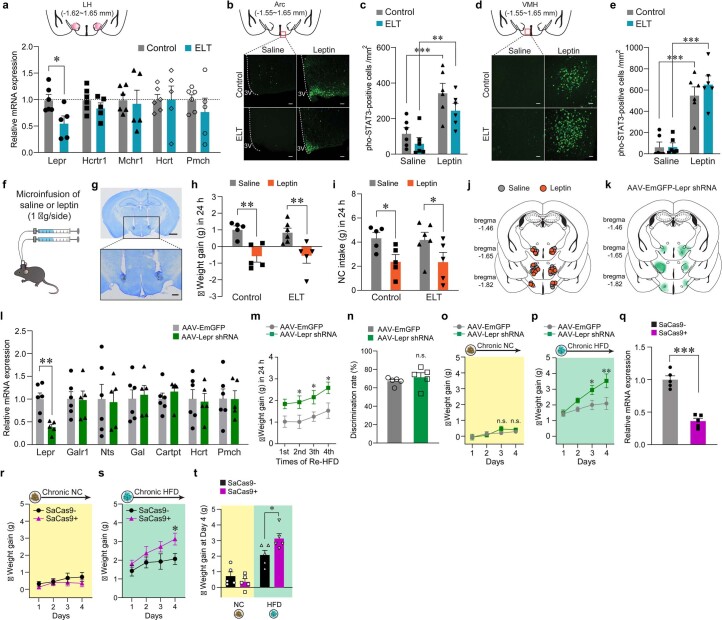
ELT downregulates Lepr signaling in the LH. (**a**) Diagram showing the dissected LH regions (top; red dashed) from adult mouse brain slices. RT-qPCR analysis for mRNA expression of Lepr, Hcrtr1, Mchr1, Hcrt and Pmch in the LH (bottom; n = 6, 5 mice per group). Two-tailed unpaired t-test, t9 = 2.749, *p = 0.0225. (**b, c**) Representative images (scale bars, 50 μm) and quantification of pSTAT3-positive cells in the Arc (n = 6 mice per group). Two-way ANOVA (F(1,20)= 29.188, p < 0.001) was followed by Fisher LSD post hoc test for multiple comparisons; **p = 0.003 compared with saline- ELT; ***p < 0.001 compared with saline- controls. (**d, e**) Representative images (scale bars, 50 μm) and quantification of pSTAT3-positive cells in the VMH (n = 6 mice per group). Two-way ANOVA (F(1,20)= 82.529, p < 0.001) was followed by Fisher LSD post hoc test for multiple comparisons; ***p < 0.001 compared with saline-treated controls or ELT mice. (**f, g**) Schematic for the microinjection of saline or leptin into the LH. Images show the location of the cannula tips in the LH. Scale bars, 1 mm (top) and 500 μm (bottom), replicated independently with similar results in five mice. (**h, i**) Microinjection of leptin (1 μg/side) into the LH reduces weight gain (**h**) and NC intake (**i**) both in control and ELT mice (n = 5, 5 for each control group; n = 6, 5 for each ELT group). In h, two-way ANOVA (F(1,17)= 27.256, p < 0.001) was followed by Fisher LSD post hoc test for multiple comparisons; **p = 0.001, **p = 0.003 compared with saline-treated control or ELT mice; in i, two-way ANOVA (F(1,17)= 9.708, p = 0.006) was followed by Fisher LSD post hoc test for multiple comparisons; *p = 0.036, *p = 0.049 compared with saline-treated control or ELT mice. (**j**) Locations of the injection cannula tips in the mice included in Extended Data Fig. 3 h, i. Symbols represent the different groups: grey circle, saline; orange circle, leptin. (**k**) Summary diagram showing the coverage of Lepr shRNA viral infusion in the LH. (**l**) Selective and robust reduction of Lepr mRNA expression in the LH by AAV-Lepr targeting shRNA (n = 6, 5 mice per group). Two-tailed unpaired t-test, t9 = 3.813, **p = 0.00413. (**m**) Body weight gain over 24 h after exposure to Re-HFD (n = 6, 7 mice per group). Two-way RM ANOVA (F(3,33)=4.869, p = 0.007) was followed by Bonferroni post hoc test for multiple comparisons; *p = 0.033, *p = 0.036, *p = 0.016 compared with EmGFP mice at respective Re-HFD cycles. (**n**) Novel object recognition test (n = 5 mice per group). Two-tailed unpaired t-test, t8 = −0.814, p = 0.439. (**o, p**) Cumulative body weight gain during 4 days of ad libitum access to either NC (**o**) or HFD (**p**) (n = 7 mice per group). In o, two-way RM ANOVA (F(1,36)= 0.197, p = 0.665); in p, two-way RM ANOVA (F(1,36)= 5.886, p = 0.032) was followed by Bonferroni post hoc test for multiple comparisons; *p = 0.024, **p = 0.001 compared with EmGFP mice at the respective days. (**q-t**) CRISPR-mediated reduction of Lepr in the LH (**q**) results in developing obesity-prone characteristics under chronic HFD exposure (r-t; n = 5 mice per group). In q, two-tailed unpaired t-test, t8 = 8.588, ***p = 0.0000261; in s, two-way RM ANOVA (F(3,24)= 7.106, p = 0.001) was followed by Bonferroni post hoc test for multiple comparisons; *p = 0.026 compared with SaCas9-; in t, two-way RM ANOVA (F(1,8)= 13.287, p = 0.007) was followed by Bonferroni post hoc test for multiple comparisons; *p = 0.018, compared with SaCas9- during HFD exposure. n.s., not significant. Data are presented as mean ± SEM.

The LH contains distinct cell types that utilize various neuromodulators to control feeding behaviors and stress responses^22,24^. Yet, it is unclear whether ELT affects neuromodulatory signaling in the LH to mediate the abnormal eating habits associated with BED and obesity. We therefore performed quantitative PCR with reverse transcription (RT–qPCR) to compare mRNA expression levels of several neuropeptides/hormones or receptors in the LH of ELT and control mice. Interestingly, Lepr mRNA expression in the LH of ELT mice was reduced compared to controls. There were no changes in other LH neuropeptides, such as hypocretin (Hcrt) or pro-melanin concentrating hormone (Pmch) or in their respective receptors (Extended Data Fig. 3a).

**Fig. 2:**
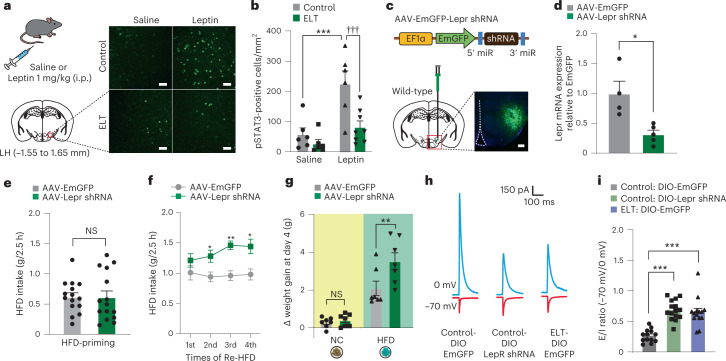
shRNA-mediated knockdown of Lepr in the LH recapitulates behavioral and electrophysiological properties of ELT mice. **a**, Schematic for the systemic injection of saline or leptin (1 mg per kg body weight, i.p.). Representative images of pSTAT3 immunostaining in the LH. Scale bars, 40 μm. **b**, Quantification of pSTAT3-positive cells in the LH (*n* = 6, 6 mice for each saline-treated group; *n* = 6, 8 for each leptin-treated group). Two-way ANOVA (*F*_(1,22) _= 11.855, *P* = 0.002) was followed by Fisher’s LSD post hoc test for multiple comparisons; ***P < 0.001 compared with saline controls; ^†††^*P* < 0.001 compared with leptin controls. **c**, Schematic for the injection of AAV-EmGFP-Lepr shRNA into the LH. Representative images of Lepr shRNA in the LH, replicated independently with similar results in 3 mice. Scale bar, 250 μm. **d**, RT–qPCR analysis of Lepr mRNA expression from the LH (*n* = 4, 5 mice per group). Two-tailed unpaired *t*-test, *t*_7_ = 3.438, *P = 0.011. **e**, 2.5 h HFD consumption during HFD priming (*n* = 15 mice per group). Two-tailed unpaired *t*-test, *t*_28_ = 0.0366, *P* = 0.971. **f**, 2.5 h HFD consumption in response to Re-HFD (*n* = 6, 7 mice per group). Two-way RM ANOVA (*F*_(1,33) _= 29.473, *P* < 0.001) was followed by Bonferroni post hoc test for multiple comparisons; *P = 0.022, **P = 0.001 and *P = 0.017, compared with EmGFP at respective Re-HFD cycles. **g**, Cumulative body weight gain during 4 d of ad libitum access to either NC or HFD (*n* = 7 mice per group). Two-way ANOVA (*F*_(1,24) _= 6.618, *P* = 0.017) was followed by Fisher’s LSD post hoc test for multiple comparisons; *P* = 0.791 compared with NC-EmGFP; **P = 0.003 compared with HFD-EmGFP. **h**,**i**, Representative traces (**h**) and quantification (**i**) of synaptic E/I ratio recorded from LH^Lepr^ neurons (*n* = 13 cells from five DIO-EmGFP-control Lepr-Cre mice, *n* = 15 cells from five DIO-Lepr shRNA control Lepr-Cre mice, and *n* = 14 cells from five DIO-EmGFP-ELT Lepr-Cre mice). One-way ANOVA (*F*_(2,39) _= 24.272, *P* < 0.001) was followed by Fisher’s LSD post hoc test for multiple comparisons; ***P < 0.001 compared with DIO-EmGFP controls. Data are the mean ± s.e.m.

Moreover, phosphorylation of STAT3 (pSTAT3, a downstream effector of Lepr)^37^ was reduced in the LH of ELT mice following systemic intraperitoneal (i.p.) administration of leptin (1 mg per kg body weight), whereas controls showed robust elevation in pSTAT3 at the same condition (Fig. 2a,b). This reduction of pSTAT3 in ELT mice was not observed in other hypothalamic subregions including the Arc or VMH (Extended Data Fig. 3b–e). These data suggest that the LH may mediate ELT-induced reduction in Lepr levels of function. To examine whether the reduced Lepr signaling of ELT mice is still functional in the LH, we injected an excessive amount of leptin (1 µg per side) into the LH and monitored NC intake and body weight. Upon reexposure to NC after 5 h of food deprivation, leptin-treated ELT mice showed reduced weight gain and food intake compared to saline-treated ELT mice (Extended Data Fig. 3f–j). Despite the decreased Lepr signaling in the LH after ELT, exogenously administered leptin can have anorexic effects, suggesting that the remaining Lepr signaling in ELT mice may mediate the physiological function of leptin.

Because Lepr signaling is important for suppressing appetite and weight gain^16^, we reasoned that selective downregulation of Lepr signaling in the LH may recapitulate the behavioral phenotypes of ELT mice, including sustained binge-like eating behaviors and enhanced vulnerability to HFD-induced obesity. To test this idea, we injected an adeno-associated virus (AAV) carrying a short hairpin RNA (shRNA) against Lepr (AAV-EmGFP-Lepr shRNA) into the LH and confirmed a robust reduction in Lepr mRNA expression without accompanying changes in the galanin receptor 1 (Galr1), neurotensin (Nts), galanin (Gal), cocaine-regulated and amphetamine-regulated transcript (Cartpt), Hcrt and Pmch in the virus injection area (Fig. 2c,d and Extended Data Fig. 3k,l).

At the HFD-priming stage, the first exposure to HFD, knockdown of Lepr in the LH did not affect HFD intake (Fig. 2e). However, in response to repeated cycles of Re-HFD, mice expressing Lepr shRNA in the LH showed augmented and sustained binge-like eating accompanied by increased weight gain (Fig. 2f and Extended Data Fig. 3m). Notably, knockdown of Lepr in the LH did not induce any differences in the ability to recognize a novel object (Extended Data Fig. 3n), suggesting that the maladaptive eating habits observed in Lepr shRNA-expressing mice cannot be ascribed to a different perception of novelty for the Re-HFD during repetitive cycles of intermittent access.

Likewise, mice expressing Lepr shRNA in the LH showed accelerated weight gain under chronic exposure to HFD but not NC (Fig. 2g and Extended Data Fig. 3o,p). We confirmed this result using a CRISPR–SaCas9 viral-based system by co-injection of two AAVs: one carrying a single-guide RNA (sgRNA) targeting the mouse Lepr locus (AAV-sgLepr^38^; a gift from D. Kong) and another expressing *Staphylococcus aureus* Cas9 (SaCas9; AAV-hSyn-SaCas9-U6-sgRNA) into the LH of wild-type mice. Under chronic HFD feeding, CRISPR-mediated reduction of Lepr in the LH increased body weight gain, whereas both groups of mice showed regular body weight gain under chronic NC feeding (Extended Data Fig. 3q–t). These data demonstrate that the CRISPR-mediated reduction of Lepr in the LH plays a significant role in enhancing vulnerability to HFD-induced obesity, consistent with our previous data using AAV shRNA-mediated Lepr knockdown.

**Extended Data Fig. 4:**
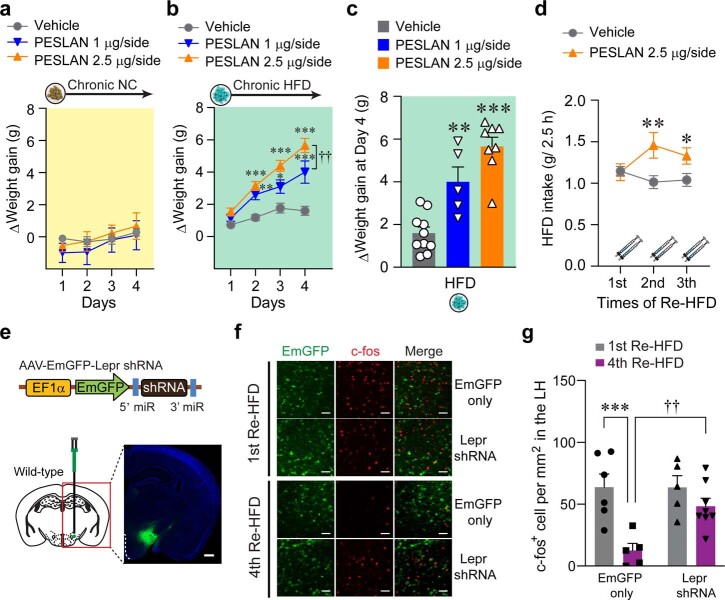
Impaired Lepr signaling in the LH recapitulates phenotypes of ELT mice. (**a, b**) Cumulative body weight gain during 4 days of ad libitum access to either NC (**a**) or HFD (**b**) after daily administrations of vehicle or PESLAN (1 or 2.5 μg/side) into the LH (n = 10, 5 and 8 mice per group). Two-way RM ANOVA (F(6,60) = 17.571, p < 0.001) was followed by Bonferroni post hoc test for multiple comparisons; *p = 0.031, **p = 0.020, ***p < 0.001 compared with vehicle group at the respective days; ††p = 0.009 for comparisons between low and high dose of PESLAN-treated mice. (**c**) Daily microinjection of PESLAN into the LH increases body weight gain on Day 4 of ad libitum access to HFD (n = 10, 5 and 8 mice per group). One-way ANOVA (F(2, 11) = 25.763, p < 0.001) was followed by Fisher LSD post hoc test for multiple comparisons; **p = 0.003, ***p < 0.001 compared with vehicle group. (**d**) 2.5 h Re-HFD consumption after microinjection of vehicle or PESLAN (2.5 μg/side) into the LH (n = 8, 7 mice per group). Two-way RM ANOVA (F(2,26) = 4.469, p = 0.021) was followed by Bonferroni post hoc test for multiple comparisons; **p = 0.003, *p = 0.041 compared with vehicle group at respective Re-HFD cycles. (**e**) Schematics depicting the injection of AAV expressing Lepr shRNA into the LH, replicated independently with similar results in five mice. Scale bar, 500 μm. (**f**) Example images of the LH showing c-fos staining in EmGFP only- or Lepr shRNA-expressing mice following the 1^st^ and 4^th^ Re-HFD. Scale bars, 50 μm. (**g**) Quantification of c-fos-positive cells in the LH of mice expressing EmGFP only or Lepr shRNA (n = 6, 5 mice for each 1^st^ Re-HFD group and n = 5, 8 mice for each 4^th^ Re-HFD group). Two-way ANOVA (F(1,20)= 15.574, p < 0.001) was followed by Fisher LSD post hoc test for multiple comparisons; ***p < 0.001 compared with EmGFP in the 1^st^ Re-HFD; ††p = 0.006 compared with EmGFP in the 4^th^ Re-HFD. Data are presented as mean ± SEM.

In addition to virus-mediated Lepr knockdown, we tested whether pharmacological inhibition of Lepr signaling in the LH increases the risk of binge-like eating and obesity following HFD exposures. We performed an intracranial microinfusion of either vehicle or pegylated superactive mouse leptin antagonist^39^ (PESLAN, p.Asp23Leu/p.Leu39Ala/p.Asp40Ala/p.Phe41Ala mutant; 1 or 2.5 μg per side) into the LH. Daily local infusion of PESLAN over 5 d increased body weight gain in a dose-dependent manner when mice were fed a HFD ad libitum, while mice in all groups showed regular body weight gain under the NC exposure (Extended Data Fig. 4a–c). Moreover, a high dose of PESLAN (2.5 μg per side) induced augmented and sustained binge-like eating in response to repeated cycles of Re-HFD (Extended Data Fig. 4d). This suggests that the pharmacological inhibition of Lepr in the LH also aggravates HFD-induced body weight gain and binge-like eating behaviors, supporting our previous observations using virus-mediated Lepr knockdown.

Furthermore, the shRNA-mediated knockdown of Lepr in the LH increased c-fos expression both upon 1st and 4th Re-HFD cycle, while mice injected with a control virus (AAV-EmGFP) showed increased c-fos induction after the 1st Re-HFD cycle but not 4th cycle (Extended Data Fig. 4e–g). Taken together, these data suggest that the reduction of Lepr signaling in the LH plays a substantial role in eliciting the binge-like eating habits and tendency toward HFD-induced obesity as well as the consistent enhancement in c-fos expression over the multiple Re-HFD cycles, mirroring the various features we observed in ELT mice.

### Roles of Lepr in the modulation of lateral hypothalamus neuronal activity

**Extended Data Fig. 5:**
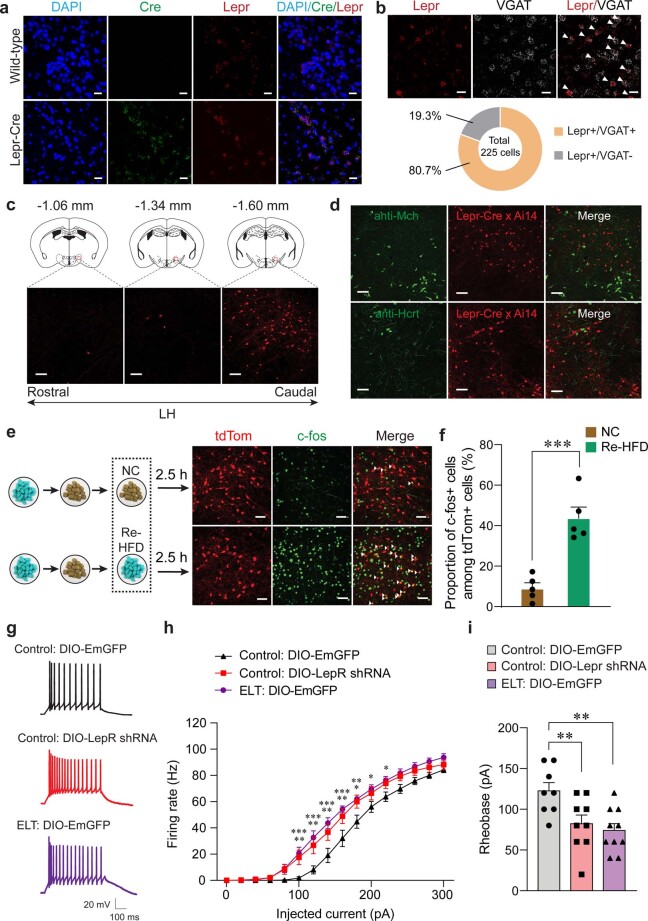
Validation of Cre expression in the LH of Lepr-Cre mice and anatomical/electrophysiological characterization of LH^Lepr^ neurons. (**a**) Representative images of Cre (green) and Lepr mRNA (red) expression in the LH of wild-type (top) or Lepr-Cre mice (bottom), replicated independently with similar results in four mice. Scale bars, 20 μm. (**b**) Representative images of Lepr (red) and VGAT mRNA (white) in the LH. Scale bars, 20 μm. Pie charts indicate percentage of LH^Lepr^ cells either colocalizing or non-colocalizing with probes to VGAT (n = 225 cells from three mice). (**c**) Lepr expression in the LH of Lepr-Cre x Ai14 mice along the rostral-caudal axis, replicated independently with similar results in three mice. Scale bars, 50 μm. (**d**) Representative image of the LH of Lepr-Cre x Ai14 mice (tdTom, red), with either Mch or Hcrt immunostaining (green), replicated independently with similar results in three mice. Scale bars, 50 μm. (**e**) Representative images showing tdTom (red) and c-fos immunoreactivity (green) in the LH of HFD-primed Lepr-Cre x Ai14 mice following exposure to either NC or Re-HFD. White arrowheads indicate the colocalization, replicated independently with similar results in five mice. Scale bars, 50 μm. (**f**) Quantification of the proportion of c-fos-positive cells among tdTom-expressing LH^Lepr^ neurons in response to NC or Re-HFD (n = 5 mice per group). Two-tailed unpaired t-test, t8 = −5.793, ***p = 0.000408. (**g**) Sample traces of neuronal firing in response to 150 pA current injections (500 ms) in LH^Lepr^ neurons of control Lepr-Cre mice expressing DIO-EmGFP (control: DIO-EmGFP), control Lepr-Cre mice expressing DIO-Lepr shRNA (control: DIO-Lepr shRNA) and ELT Lepr-Cre mice expressing DIO-EmGFP (ELT: DIO-EmGFP). (**h**) Action potential firing rate in response to increasing current injection steps in LH^Lepr^ neurons (n = 8 cells from three control: DIO-EmGFP mice, n = 9 cells from three control: DIO-Lepr shRNA mice, and n = 10 cells from three ELT: DIO-EmGFP mice). Two-way RM ANOVA (F(30,360) = 3.237, p < 0.001) was followed by Bonferroni post hoc test for multiple comparisons; at 100 pA, **p = 0.008 for control: DIO-EmGFP versus control: DIO-Lepr shRNA, ***p < 0.001 for control: DIO-EmGFP versus ELT: DIO-EmGFP; at 120 pA, **p = 0.001 for control: DIO-EmGFP versus control: DIO-Lepr shRNA, ***p < 0.001 for control: DIO-EmGFP versus ELT: DIO-EmGFP; at 140 pA, **p = 0.002 for control: DIO-EmGFP versus control: DIO-Lepr shRNA, ***p < 0.001 for control: DIO-EmGFP versus ELT: DIO-EmGFP; at 160 pA, **p = 0.004 for control: DIO-EmGFP versus control: DIO-Lepr shRNA, ***p < 0.001 for control: DIO-EmGFP versus ELT: DIO-EmGFP; at 180 pA, *p = 0.011 for control: DIO-EmGFP versus control: DIO-Lepr shRNA, **p = 0.003 for control: DIO-EmGFP versus ELT: DIO-EmGFP; at 200 pA, *p = 0.024 for control: DIO-EmGFP versus ELT: DIO-EmGFP; at 220 pA, *p = 0.049 for control: DIO-EmGFP versus ELT: DIO-EmGFP. (**i**) The rheobase measurements for LH^Lepr^ neurons from (**h**) (n = 8 cells from three control: DIO-EmGFP mice, n = 9 cells from three control: DIO-Lepr shRNA mice and n = 10 cells from three ELT: DIO-EmGFP mice). One-way ANOVA (F(2, 24) = 6.664, p = 0.005) was followed by Fisher LSD post hoc test for multiple comparisons; **p = 0.002, **p = 0.009 compared with control: DIO-EmGFP mice. Data are presented as mean ± SEM.

Lepr suppresses both appetite and weight gain, but how the downregulation of Lepr in the LH drives the neural activity change to mediate binge-like eating or obesity is unknown. We investigated this using Lepr-Cre mice. Using dual-fluorescence in situ hybridization (FISH), we confirmed that Lepr-Cre mice express Cre recombinase specifically in LH^Lepr^ neurons (Extended Data Fig. 5a). Consistent with previous reports^40,41^, we found that most LH^Lepr^ neurons (80.7%) are GABAergic (Extended Data Fig. 5b). To examine the anatomical configuration of LH^Lepr^ neurons, we crossed Lepr-Cre mice with the Cre-dependent tdTomato (tdTom) reporter Ai14 line (Lepr-Cre × Ai14 hereafter). We found that the tdTom-labeled Lepr-expressing neurons are located primarily in the caudal LH and that LH^Lepr^ neurons are distinct from the Mch-expressing or Hcrt-expressing neuronal populations (Extended Data Fig. 5c,d).

To explore the functional relevance of the LH^Lepr^ neurons in binge-like consumption, we first asked whether the Re-HFD-induced expression of c-fos occurs preferentially among LH^Lepr^ neurons. Using Lepr-Cre × Ai14 animals previously primed to a HFD, we measured the proportion of c-fos-positive cells among tdTom-expressing LH neurons following Re-HFD. The Re-HFD challenge enhanced c-fos expression in LH^Lepr^ neurons by 42% (Extended Data Fig. 5e,f), indicating that LH^Lepr^ neurons constitute a population that is preferentially activated under binge-like eating conditions.

Given that Lepr signaling modulates synaptic plasticity in hypothalamic neurons^38^, it is possible that the reduced Lepr signaling in the LH leads to binge-like eating by altering electrophysiological properties of LH^Lepr^ neurons. To test this, we injected the LH of control Lepr-Cre mice with an AAV carrying a Cre-dependent Lepr shRNA (AAV-DIO-EmGFP-Lepr shRNA) and performed whole-cell patch-clamp recordings of LH^Lepr^ neurons. We found a significant increase in the ratio of electrically evoked excitatory inputs to inhibitory inputs (E/I ratio) in Lepr shRNA-expressing neurons compared to those expressing EmGFP alone. In addition, the LH^Lepr^ neurons of ELT mice showed an increased E/I ratio (Fig. 2h,i). Moreover, LH^Lepr^ neurons from both control mice expressing Lepr shRNA and ELT mice showed increased intrinsic excitability and became more excitable (Extended Data Fig. 5g–i), suggesting that the ELT enhances the net activity of LH^Lepr^ neurons, possibly through downregulation of Lepr signaling.

### Early-life trauma LH^Lepr^ neurons are activated after repetitive reexposure to a HFD

**Fig. 3:**
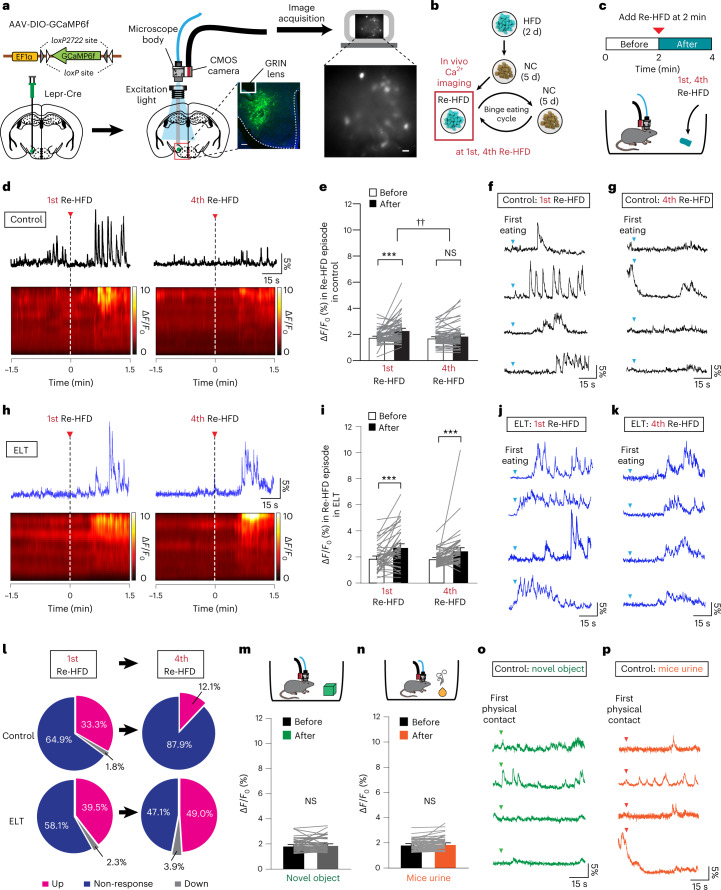
LH^Lepr^ neurons of ELT mice exhibit increased activity in response to multiple Re-HFD. **a**, Schematic for the injection of AAV expressing Cre-dependent GCaMP6f into the LH of Lepr-Cre mice. Confocal image (left) showing GRIN lens placement on the GCaMP6f-expressing LH^Lepr^ neurons, replicated independently with similar results in 5 mice. Scale bar, 250 μm. A sample image (right) of GCaMP6f-expressing LH^Lepr^ neurons. Scale bar, 25 μm. **b**,**c**, Schematics for experimental setup to record in vivo Ca^2+^ activity in GCaMP6f-expressing LH^Lepr^ neurons for 2 min before and after presenting the 1st and 4th Re-HFD. **d**, Example traces and heat maps from LH^Lepr^ neurons of controls before and after presenting the 1st and 4th Re-HFD. Red arrows and vertical dashed lines mark the time Re-HFD was introduced. **e**, Average Ca^2+^ responses of Δ*F/F*_0_ in LH^Lepr^ neurons of controls during the 1st or 4th Re-HFD cycle (*n* = 57 cells from 7 mice and 58 cells from 7 mice for each Re-HFD cycle). Two-way RM ANOVA (*F*_(1,113) _= 28.092, *P* < 0.001) was followed by Bonferroni post hoc test for multiple comparisons; ***P < 0.001 for average Δ*F/F*_0_ before versus after Re-HFD introduction during 1st Re-HFD cycle; *P* = 0.069 for average Δ*F/F*_0_ before versus after Re-HFD introduction during 4th Re-HFD cycle; ^††^*P* = 0.002 for average Δ*F/F*_0_ after Re-HFD introduction in the 1st versus 4th Re-HFD cycle. **f**,**g**, Examples of individual traces of LH^Lepr^ neuronal activity from four representative cells of controls after the first eating bout of the 1st and 4th Re-HFD. Blue arrow marks the first eating bouts. **h**, Example traces and heat maps from LH^Lepr^ neurons of ELT mice before and after presenting the 1st and 4th Re-HFD. Red arrows and vertical dashed lines mark the time Re-HFD was introduced. **i**, Average Ca^2+^ responses of Δ*F/F*_0_ in LH^Lepr^ neurons of ELT mice during the 1st or 4th Re-HFD cycle (*n* = 43 cells from 5 mice and 51 cells from 5 mice for each Re-HFD cycle). Two-way RM ANOVA (*F*_(1,92) _= 30.449, *P* < 0.001) was followed by Bonferroni post hoc test for multiple comparisons; ***P < 0.001 for average Δ*F/F*_0_ before versus after Re-HFD introduction either during 1st Re-HFD or 4th Re-HFD cycle. **j**,**k**, Example traces of LH^Lepr^ neuronal activity from four representative cells of ELT mice after the first eating bout of the 1st and 4th Re-HFD. Blue arrows mark the first eating bouts. **l**, Pie charts indicate the classification of LH^Lepr^ neurons of control or ELT mice showing upregulation, non-response or downregulation in average Ca^2+^ responses after introducing Re-HFD during the 1st and 4th cycles. **m**,**n**, Average Ca^2+^ responses of Δ*F/F*_0_ in LH^Lepr^ neurons of controls before and after the first physical contact to novel object (**m**; *n* = 51 cells from 4 mice) or mice urine from the opposite sex conspecifics (**n**; *n* = 48 cells from 4 mice). In **m**, two-tailed paired *t*-test, *t*_50_ = −0.737, *P* = 0.465; in **n**, two-tailed paired *t*-test, *t*_47_ = −1.810, *P* = 0.0767. **o**,**p**, Example traces of LH^Lepr^ neuronal activity from four representative cells of control mice after the first physical contact to novel object or mice urine. Data are the mean ± s.e.m. Δ*F/F*_0_, average cell fluorescence change.

After we found that the Lepr knockdown increases binge-like HFD consumption and the net activity of the LH^Lepr^ neurons (Fig. 2f,i), we hypothesized that endogenous LH^Lepr^ neuronal activation may encode key behavioral features of binge-like eating habits in ELT mice. Given that ELT mice showed more sustained binge-like HFD overconsumption than controls (Fig. 1d), we asked whether multiple Re-HFD exposures consistently increase LH^Lepr^ neuronal activity in ELT mice. To do this, we implanted a gradient-index (GRIN) lens-based mini-microscope above LH^Lepr^ neurons expressing the Ca^2+^ indicator GCaMP6f^42^. Six weeks after surgery, we compared the dynamics of GCaMP6 fluorescence in response to the 1st or 4th Re-HFD between control and ELT Lepr-Cre mice (Fig. 3a–c). We found that the activity of LH^Lepr^ neurons in controls was increased in response to the 1st Re-HFD, but not to the 4th Re-HFD (Fig. 3d–g). On the contrary, ELT mice increased LH^Lepr^ neuronal activity both in the 1st and 4th Re-HFD cycles (Fig. 3h–l). In the presence of non-food items, such as a novel object (for example, Lego brick) or social stimulus (for example, mice urine from the opposite sex conspecifics), we were not able to observe the increased LH^Lepr^ neuronal responses (Fig. 3m–p). These data support that LH^Lepr^ neuronal activity is more strongly correlated with the context of HFD, rather than with general novelty, motor or sensory aspects of the behavior. When we defined the upregulated cell as its average GCaMP6 fluorescence in the presence of Re-HFD is above the baseline plus the standard deviation, 33.3% of LH^Lepr^ neurons of control mice met this criterion during the 1st Re-HFD, while the decreased proportion of LH^Lepr^ neurons (12.1%) was upregulated after the 4th Re-HFD exposure. In contrast to this, ELT mice displayed more stable maintenance in the proportion of upregulated cells even after multiple exposures to Re-HFD; that is, 39.5% and 49.0% during the 1st and 4th Re-HFD stages, respectively (Fig. 3l). Together, these data support the hypothesis that the LH^Lepr^ neurons of ELT mice are consistently activated after the repeated Re-HFD exposures, which may lead to sustained binge-like eating habits.

**Extended Data Fig. 6:**
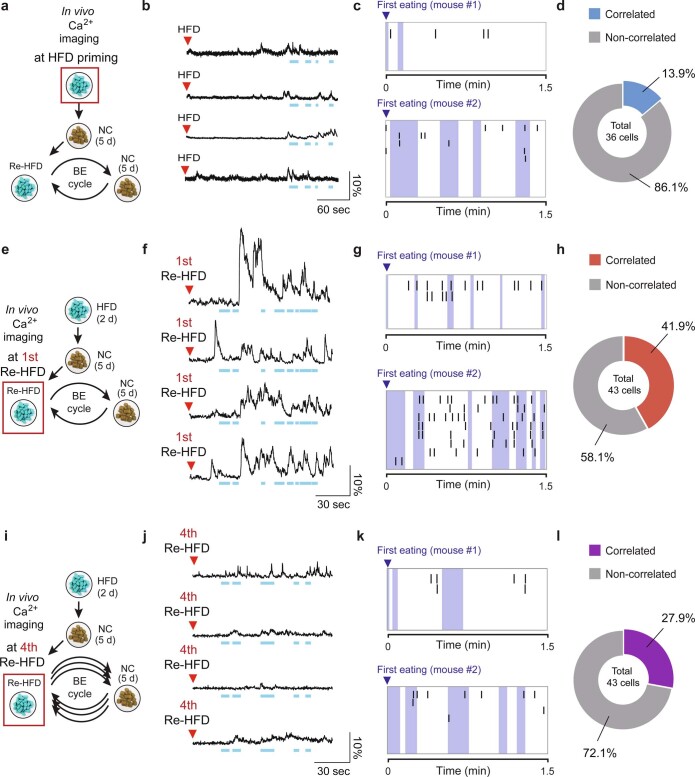
In vivo imaging of Ca^2+^ dynamics from LH^Lepr^ neurons at different HFD stages. (**a, b**) Experimental timeline and example traces of LH^Lepr^ neuronal activity from four representative cells during HFD priming stage. Blue dashed areas indicate eating bouts. (**c**) Raster plots showing LH^Lepr^ neuronal activity from two representative mice aligned to the first eating bout onset during HFD-priming. The rows and ticks in the raster plots represent individual cells and a single Ca^2+^ transient event, respectively. Blue dashed areas indicate eating bouts. (**d**) Pie charts indicate the percentage of LH^Lepr^ neurons generating Ca^2+^ transient events at the onset of eating bouts during HFD-priming stage (n = 36 cells from four mice). (**e, f**) Experimental timeline and example traces of LH^Lepr^ neuronal activity from four representative cells during the 1st Re-HFD. Blue dashed areas indicate eating bouts. (**g**) Raster plots showing LH^Lepr^ neuronal activity from two representative mice aligned to the first eating bout onset during the 1st Re-HFD. The rows and ticks in the raster plots represent individual cells and a single Ca^2+^ transient event, respectively. Blue dashed areas indicate eating bouts. (**h**) Pie charts indicate the percentage of LH^Lepr^ neurons generating Ca^2+^ transient events at the onset of eating bouts during the 1st Re-HFD stage (n = 43 cells from five mice). (**i, j**) Experimental timeline and example traces of LH^Lepr^ neuronal activity from four representative cells during the 4th Re-HFD. Blue dashed areas indicate eating bouts. (**k**) Raster plots showing LH^Lepr^ neuronal activity from two representative mice aligned to the first eating bout onset during the 4th Re-HFD. The rows and ticks in the raster plots represent individual cells and a single Ca^2+^ transient event, respectively. Blue dashed areas indicate eating bouts. (**l**) Pie charts indicate the percentage of LH^Lepr^ neurons generating Ca^2+^ transient events at the onset of eating bouts during the 4th Re-HFD stage (n = 43 cells from five mice).

To further confirm whether LH^Lepr^ neurons encode the ‘binge-like eating’ characteristics, we asked how well LH^Lepr^ neuronal activity is correlated with eating bouts during Re-HFD exposures compared to ones during HFD priming. Using control Lepr-Cre mice, we extracted in vivo Ca^2+^ transients and quantified the proportion of LH^Lepr^ neurons that produce the Ca^2+^ transients at the onset of eating bouts. During the HFD priming, we observed that only 13.9% of LH^Lepr^ neurons generate Ca^2+^ transients correlated with eating bouts (Extended Data Fig. 6a–d). However, higher proportions of LH^Lepr^ neurons in the 1st Re-HFD (41.9%) and, to a lesser extent, in the 4th Re-HFD stage (27.9%) were responsive to eating bouts (Extended Data Fig. 6e–l). These data suggest that LH^Lepr^ neuronal activity is more correlated with the stage-specific binge-like HFD consumption upon Re-HFD exposures than with general consummatory behaviors.

### Projection-specific roles of LH^Lepr^ in binge-like eating

**Extended Data Fig. 7:**
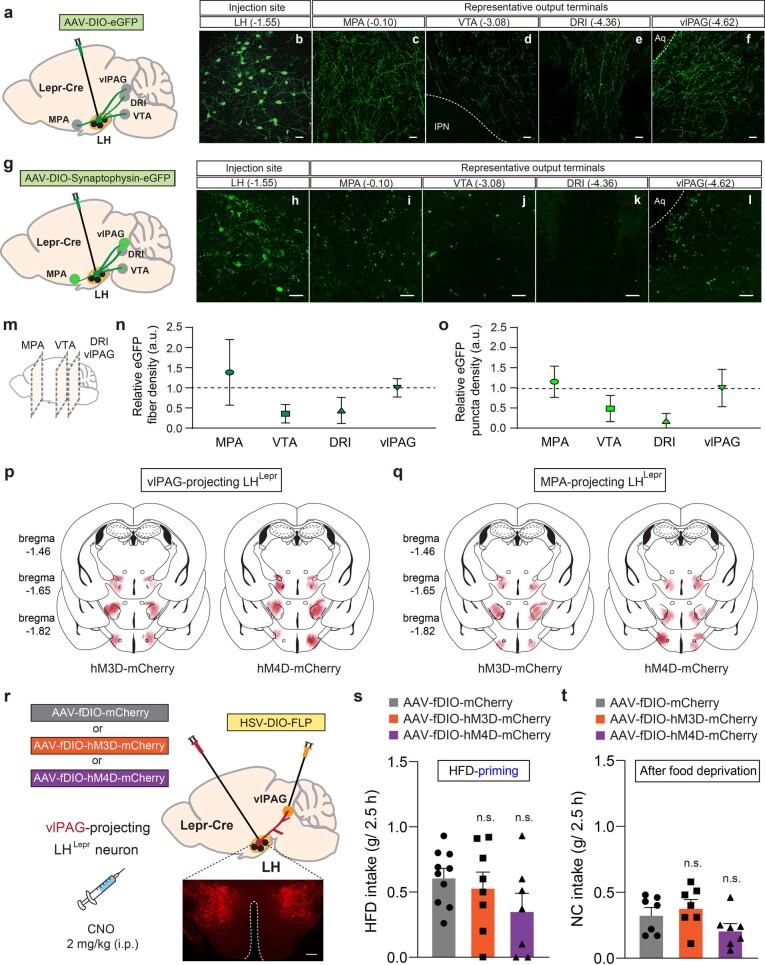
Modulation of LH^Lepr^→ vlPAG neuronal activity does not affect general food intake. (**a**) Schematic for the injection of AAV expressing Cre-dependent eGFP into the LH of Lepr-Cre mice. (**b-f**) Representative images showing eGFP expression in the LH^Lepr^ neuronal cell bodies (**b**) and axons in the MPA (**c**), VTA (**d**), DRI (**e**) and vlPAG (**f**), replicated independently with similar results in four mice. IPN, interpeduncular nucleus; Aq, aqueduct. Scale bar, 50 μm. (**g**) Schematic for the injection of AAV expressing Cre-dependent synaptophysin-eGFP into the LH of Lepr-Cre mice. (**h-l**) Representative images showing synaptophysin-eGFP expression in the LH^Lepr^ neuronal cell bodies (**h**) and synaptic puncta in the MPA (**i**), VTA (**j**), DRI (**k**) and vlPAG (**l**), replicated independently with similar results in four mice. Scale bars, 40 μm. (**m**) Sagittal view showing areas along antero-posterior axis images are taken for fiber or synaptic puncta quantitation analysis. (**n, o**) Quantification of fiber density (n; n = 4 mice per group) and synaptophysin-eGFP puncta density (o; n = 4 mice per group) in downstream targets of LH^Lepr^ neurons. (**p, q**) Summary diagram showing the coverage of hM3D-mCherry (left), hM4D-mCherry (right) viral infusion in the LH^Lepr^ → vlPAG neurons (**p**) and LH^Lepr^ → MPA neurons (**q**) in Lepr-Cre mice. (**r**) Schematic depicting the viral injection for chemogenetic manipulation of LH^Lepr^→ vlPAG neurons in control Lepr-Cre mice. Confocal image showing mCherry-expressing LH^Lepr^ neurons, replicated independently with similar results in four mice. Scale bar, 500 μm. (**s**) 2.5 h HFD consumption at the priming stage with CNO-induced activation or inhibition of LH^Lepr^→ vlPAG neurons (n = 10, 8 and 7 mice for each group). One-way ANOVA (F(2,22)= 1.603, p = 0.224). (**t**) 2.5 h NC consumption after 5 h food deprivation with CNO-induced activation or inhibition of LH^Lepr^→ vlPAG neurons (n = 7 mice per group). One-way ANOVA (F(2,18)= 2.634, p = 0.099). n.s., not significant. Data are presented as mean ± SEM.

Based on our observation of consistent activation of LH^Lepr^ neurons in ELT mice upon repeated Re-HFD exposures (Fig. 3p), we hypothesized that selective inhibition of LH^Lepr^ neurons may normalize the pathological binge-like eating habits and tendency toward HFD-induced obesity associated with ELT. Given the previous studies indicating that the LH^Lepr^ neurons project to multiple downstream structures involved in various behaviors^40,43^, it is plausible that the downstream target-specific LH^Lepr^ neurons mediate ELT-induced abnormal eating habits. We, therefore, delineated the efferent connections of LH^Lepr^ neurons by injecting an AAV expressing eGFP in a Cre-dependent manner (AAV-DIO-eGFP) into the LH of Lepr-Cre mice (Extended Data Fig. 7a). We observed eGFP-labeled LH^Lepr^ neuronal cell bodies (Extended Data Fig. 7b) and axonal fibers in several brain areas, including the MPA, the ventral tegmental area (VTA), the interfascicular regions of the dorsal raphe (DRI) and the vlPAG (Extended Data Fig. 7c–f). We also evaluated the synaptic targets of those projections by injecting AAV-DIO-synaptophysin-eGFP into the LH of Lepr-Cre mice (Extended Data Fig. 7g,h), which enables labeling of LH^Lepr^ presynaptic terminals. We observed only a few eGFP-labeled synaptic puncta in the DRI, but relatively dense innervations in the MPA, VTA and vlPAG (Extended Data Fig. 7i–l). Together, these data suggest that LH^Lepr^ neurons send projections to multiple brain structures but form synapses primarily with the three downstream areas, such as the MPA, VTA and vlPAG (Extended Data Fig. 7m–o).

**Fig. 4:**
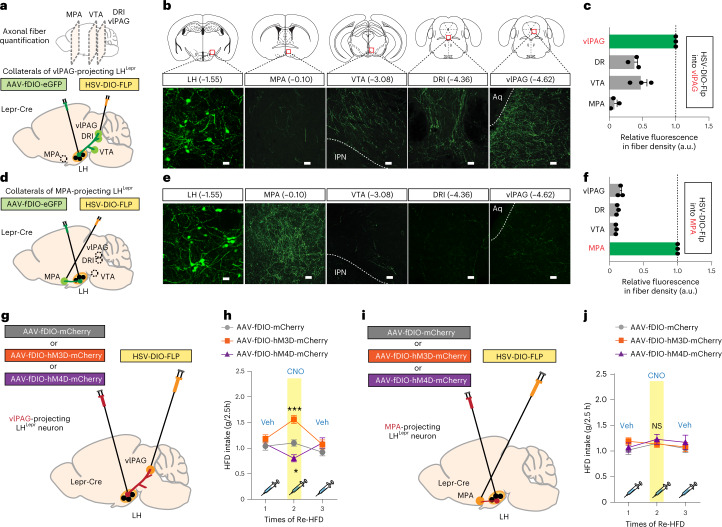
Modulation of LH^Lepr ^→ vlPAG neuronal activity influences binge-like eating. **a**, Images showing a sagittal view along the anteroposterior axis were taken for fiber quantitation analysis. Schematic depicting viral strategy with the injection of HSV-DIO-Flp into the vlPAG and AAV-fDIO-eGFP into the LH of Lepr-Cre mice. **b**, Coronal views of the areas in which confocal images were taken (red squares). Representative images of cell bodies in the LH (scale bar, 25 μm) and fibers in target areas including the MPA, VTA, DRI and vlPAG (scale bars, 50 μm). IPN, interpeduncular nucleus; Aq, aqueduct. **c**, Fiber quantification of LH^Lepr ^→ vlPAG neurons. Dashed line indicates normalized level of eGFP-labeled fiber density (*n* = 3 control Lepr-Cre mice). **d**, Schematic depicting viral strategy with the injection of HSV-DIO-Flp into the MPA and AAV-fDIO-eGFP into the LH of Lepr-Cre mice. **e**, Representative confocal images of cell bodies in the LH (scale bar, 25 μm) and fibers in target areas including the MPA, VTA, DRI and vlPAG (scale bars, 50 μm). **f**, Fiber quantification of LH^Lepr ^→ MPA neurons. Dashed line indicates normalized level of eGFP-labeled fiber density (*n* = 3 control Lepr-Cre mice). **g**, Schematic depicting the viral injection for chemogenetic manipulation of LH^Lepr ^→ vlPAG neurons in control Lepr-Cre mice. **h**, CNO-induced activation or inhibition of LH^Lepr ^→ vlPAG neurons aggravated or alleviated binge-like HFD consumption, respectively (*n* = 7, 8 and 5 mice for each group). Two-way RM ANOVA (*F*_(4,34) _= 6.079, *P* < 0.001) was followed by Bonferroni post hoc test for multiple comparisons; ***P < 0.001 and *P = 0.02, compared with mice expressing fDIO-mCherry alone in the presence of CNO. **i**, Schematic depicting the viral injection for chemogenetic manipulation of LH^Lepr ^→ MPA neurons in control Lepr-Cre mice. **j**, Chemogenetic modulation of LH^Lepr ^→ MPA neurons did not affect binge-like HFD consumption (*n* = 6, 5 and 6 mice for each group). Two-way RM ANOVA (*F*_(2,29) _= 0.354, *P* = 0.708). Data are the mean ± s.e.m. a.u., arbitrary units.

These findings, however, do not distinguish whether individual LH^Lepr^ neurons send collateralized axons to multiple target structures or whether the LH^Lepr^ neurons projecting to each downstream target represent distinct populations. To investigate this, we first examined two main LH^Lepr^ neuronal targets, the vlPAG and MPA, which show significant amounts of LH^Lepr^ axonal fibers and synaptic terminals (Extended Data Fig. 7m–o). We injected retrogradely transported herpes simplex virus (HSV) expressing Flp recombinase in a Cre-dependent manner (HSV-DIO-Flp) into either the vlPAG or the MPA concurrently with injections of an AAV expressing a Flp-dependent eGFP (AAV-fDIO-eGFP) into the LH of control Lepr-Cre mice (Fig. 4a,d). In this manner, only the LH^Lepr^ neurons that project to the injection site of the HSV-DIO-Flp (that is, vlPAG or MPA) will be labeled. The vlPAG-projecting LH^Lepr^ (LH^Lepr ^→ vlPAG) neurons sent collateralized axons to the VTA, DRI and, to a lesser extent, MPA (Fig. 4b,c). MPA-projecting LH^Lepr^ (LH^Lepr ^→ MPA) neurons, however, project predominantly to the MPA with a little portion of collateralized axons into other target areas (Fig. 4e,f), suggesting that LH^Lepr ^ → vlPAG and LH^Lepr ^→ MPA neurons represent mostly distinct neuronal populations.

Next, we sought to examine whether these two discrete circuits—LH^Lep r^→ vlPAG and LH^Lepr ^→ MPA—make different contributions to binge-like eating behaviors. We took a chemogenetic approach by using designer receptors exclusively activated by designer drugs (DREADDs)^44^. We expressed the Gq-coupled or Gi-coupled DREADDs hM3Dq or hM4Di in the LH^Lepr ^→ vlPAG neurons by injecting HSV-DIO-Flp into the vlPAG and AAV-fDIO-hM3Dq-mCherry, AAV-fDIO-hM4Di-mCherry or AAV-fDIO-mCherry bilaterally into the LH of control Lepr-Cre mice (Fig. 4g and Extended Data Fig. 7p). This allowed us to selectively activate or inhibit LH^Lepr ^→ vlPAG neurons in the presence of clozapine-*N*-oxide (CNO), an inert ligand specific to the hM3Dq and hM4Di receptors. During repeated cycles of Re-HFD, CNO-mediated activation of LH^Lepr ^→ vlPAG neurons significantly increased HFD consumption (g/2.5 h), whereas CNO-mediated inhibition of LH^Lepr ^→ vlPAG neurons attenuated it (Fig. 4h). In contrast, the similar manipulation of LH^Lepr ^→ MPA neurons did not affect binge-like eating (Fig. 4i,j and Extended Data Fig. 7q). These data suggest that LH^Lepr ^→ vlPAG neurons play a more important role in regulating binge-like eating than LH^Lepr ^→ MPA neurons.

To further confirm the specific contribution of LH^Lepr ^→ vlPAG neurons to binge-like HFD consumption, we monitored HFD intake at the priming stage or NC intake following mild food deprivation. In both cases, CNO-mediated activation or inhibition of LH^Lepr ^→ vlPAG neuronal activity did not change food intake (Extended Data Fig. 7r–t). This suggests that the LH^Lepr ^→ vlPAG circuit specifically encodes HFD consumption in the context of binge-like eating, independent of other general food intakes such as HFD consumption during the priming stage or physical hunger-induced NC consumption.

**Fig. 5:**
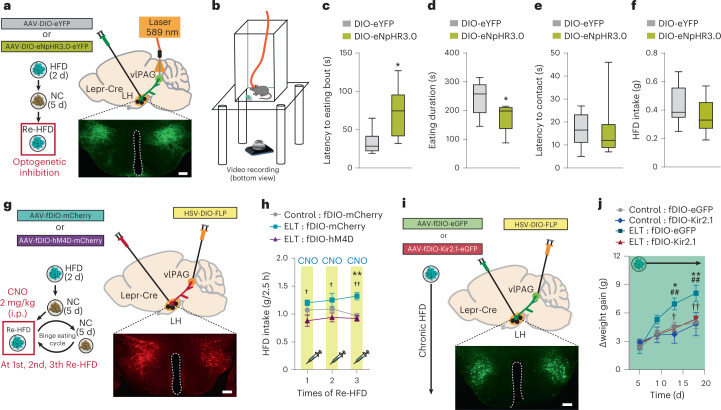
Inhibition of LH^Lepr ^→ vlPAG neurons prevents early-life trauma-induced binge-like eating and obesity. **a**, Schematic for photoinhibition of LH^Lepr ^→ vlPAG neurons in control Lepr-Cre mice. Confocal image showing eNpHR3.0-eYFP-expressing LH^Lepr^ neurons, replicated independently with similar results in 8 mice. Scale bar, 500 μm. **b**, Schematic for the setup of a bottom-view video recording for monitoring binge-like consumption. **c**–**f**, Photoinhibition of LH^Lepr^ → vlPAG neurons in control mice increases latency to the first eating bout (**c**) and reduces eating duration (**d**) with no significant changes in the latency to physical contact of the food (**e**) and total HFD intake over 30 min (**f**). Box plots display the median (center) and 2.5th to 97.5th percentiles of the distribution (bounds) with whiskers extending from minimum to maximum values (*n* = 6, 8 mice per group). Two-tailed unpaired *t*-test; in **c**, *t*_12_ = −2.671, *P = 0.0204; in **d**, *t*_12_ = 2.416, *P = 0.0326; in **e**, *t*_12_ = 0.0706, *P* = 0.945; in **f**, *t*_12_ = 1.042, *P* = 0.318. **g**, Schematic for chemogenetic inhibition of LH^Lepr ^→ vlPAG neurons of control or ELT Lepr-Cre mice. Confocal image showing hM4D-mCherry-expressing LH^Lepr^ neurons, replicated independently with similar results in 5 mice. Scale bar, 500 μm. **h**, CNO-mediated inhibition of LH^Lepr^ → vlPAG neurons normalized augmented and sustained binge-like eating in ELT mice upon Re-HFD access (*n* = 6, 5 and 5 mice per group). Two-way RM ANOVA (*F*_(2,26) _= 12.134, *P* = 0.001) was followed by Bonferroni post hoc test for multiple comparisons; ^†^*P* = 0.010, ^†^*P* = 0.014 and ^††^*P* = 0.002, compared with fDIO-hM4D-expressing ELT mice at respective Re-HFD cycles; **P = 0.002 compared with fDIO-mCherry-expressing control mice at the respective Re-HFD cycle. **i**, Schematic depicting the expression of either control virus (eGFP) or Kir2.1 in LH^Lepr ^→ vlPAG neurons of control or ELT Lepr-Cre mice. Confocal image showing Kir2.1-eGFP expressing LH^Lepr^ neurons, replicated independently with similar results in 6 mice. Scale bar, 500 μm. **j**, Kir2.1-mediated silencing of LH^Lepr ^→ vlPAG neurons normalized the HFD-induced obesity in ELT mice (*n* = 7, 4 mice for control mice expressing either eGFP alone or Kir2.1, respectively; *n* = 5, 6 mice for ELT mice expressing either eGFP alone or Kir2.1, respectively). Two-way RM ANOVA (*F*_(9,54) _= 3.912, *P* < 0.001) was followed by Bonferroni post hoc test for multiple comparisons; ^†^*P* = 0.014 and ^††^*P* = 0.009, for comparisons between ELT: fDIO-eGFP versus ELT: fDIO-Kir2.1 at respective days; *P = 0.021 and **P = 0.002, for comparisons between ELT: fDIO-eGFP versus control: fDIO-eGFP at respective days; ^##^*P* = 0.005 and ^##^*P* = 0.004, for comparisons between ELT-fDIO-eGFP versus control: fDIO-Kir2.1 at respective days. Data are the mean ± s.e.m.

**Extended Data Fig. 8:**
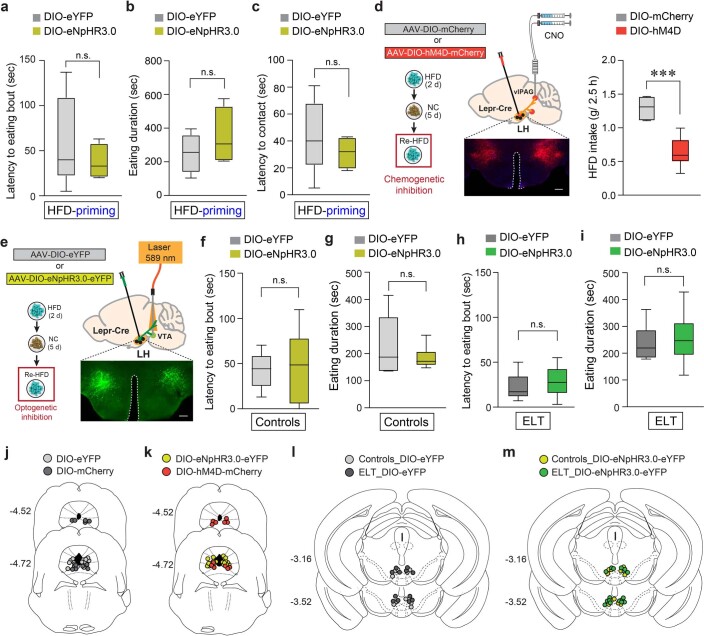
Inhibition of LH^Lepr^→ VTA neurons does not affect binge-like eating. (**a-c**) Photoinhibition of LH^Lepr^→ vlPAG neurons in controls during the HFD priming. Box plots displaying median (center) and 2.5 to 97.5 percentiles of the distribution (bound) with whiskers from minimum to maximum value (n = 5, 4 mice per group). Two-tailed unpaired t-test; in a, t7 = 0.854, p = 0.421; in b, t7 = −1.012, p = 0.345; in c, t7 = 0.847, p = 0.425. (**d**) Schematic for target-specific chemogenetic manipulation in LH^Lepr^→ vlPAG neurons of controls. Image showing hM4D-mCherry-expressing LH^Lepr^ neurons. Scale bar, 500 μm. Box plots displaying median (center) and 2.5 to 97.5 percentiles of the distribution (bound) with whiskers from minimum to maximum value (n = 6 mice per group). Two-tailed unpaired t-test; t10 = 5.777, ***p = 0.000178. (**e**) Schematic for optogenetic inhibition of LH^Lepr^→ VTA neurons. Confocal image showing eNpHR3.0-eYFP-expressing LH^Lepr^, replicated independently with similar results in five mice. Scale bar, 500 μm. (**f-i**) No effects of optogenetic inhibition of LH^Lepr^→ VTA neurons of controls or ELT mice on behavioral features of binge-like HFD eating (in f, h, latency to first eating bout; in g, i, eating duration). Box plots displaying median (center) and 2.5 to 97.5 percentiles of the distribution (bound) with whiskers from minimum to maximum value (n = 5, 6 mice per group). Two-tailed unpaired t-test; in f, t9 = −0.223, p = 0.828; in g, t9 = 0.807, p = 0.440; in h, t12 = −0.719, p = 0.486; in i, t12 = −0.392, p = 0.702. (**j, k**) Locations of the optic fibers or guide cannula included in Fig. 5c-f, Extended Data Fig. 8d, respectively. Symbols represent the different groups: light grey circle, AAV-DIO-eYFP; dark grey circle, AAV-DIO-mCherry; light green circle, AAV-DIO-eNpHR3.0-eYFP; orange circle, AAV-DIO-hM4D-mCherry. (**l, m**) Locations of the optic fibers included in Extended Data Fig. 8f-i. Symbols represent the different groups: light grey circle, control mice with AAV-DIO-eYFP; dark grey circle, ELT mice with AAV-DIO-eYFP; light green circle, control mice with AAV-DIO-eNpHR3.0-eYFP; dark green circle, ELT mice with AAV-DIO-eNpHR3.0-eYFP. n.s., not significant. Data are presented as mean ± SEM.

Our anatomical tracing study revealed collateral projections from LH^Lepr ^→ vlPAG neurons into the VTA (Fig. 4c), suggesting the coordinated regulation of LH^Lepr ^→ VTA neuronal activity. To discriminate the functional relevance of LH^Lepr^ → vlPAG and LH^Lepr ^→ VTA neurons in binge-like eating, we used optogenetic terminal inhibition. We injected either AAV-DIO-eNpHR3.0-eYFP or AAV-DIO-eYFP into the LH of control Lepr-Cre mice and then implanted optic cannulae over the vlPAG (Fig. 5a). We subjected mice to Re-HFD during illumination with 589 nm of light and monitored binge-like eating behaviors (Fig. 5b). Interestingly, mice with silencing of LH^Lepr ^→ vlPAG neuronal terminals showed slower responses in their first eating bout and less time spent in eating, but similar latency in physical approach to the food and total intake level (Fig. 5c–f and Extended Data Fig. 8j,k). However, during HFD priming, the same optogenetic inhibition of LH^Lepr^ → vlPAG neuronal terminals did not change those behavioral features (Extended Data Fig. 8a–c).

Because recent studies have challenged the efficacy of optogenetic terminal inhibition^45^, we looked to further confirm our optogenetic results. Utilizing target-specific inhibition of LH^Lepr^ neuronal projections expressing Gi DREADD by the local infusion of CNO into the vlPAG, we confirmed that inhibiting LH^Lepr^ neuronal terminals in the vlPAG significantly reduced HFD intake (g/2.5 h) in response to Re-HFD (Extended Data Fig. 8d,j,k), supporting the optogenetic manipulation data. Together, these data indicate that reducing LH^Lepr^ → vlPAG neuronal activity selectively alleviates binge-like HFD consumption, instead of changing general eating behaviors toward HFD initially given.

On the other hand, we confirmed that eNpHR3.0-mediated inhibition of LH^Lepr^ → VTA neuronal terminals in control Lepr-Cre mice affected neither latency to the first eating bout nor total eating duration in response to Re-HFD (Extended Data Fig. 8e–g,l,m). Likewise, in ELT Lepr-Cre mice, no difference was seen in those behavioral features during binge-like HFD consumption after eNpHR3.0-mediated inhibition of LH^Lepr^ → VTA neuronal terminals (Extended Data Fig. 8h,i,l,m). These data support the hypothesis that increased activity of LH^Lepr ^→ vlPAG, but not LH^Lepr ^→ VTA neurons, is necessary to encode the key behavioral features of binge-like HFD consumption.

### Inhibition of LH^Lepr ^→ vlPAG reverses binge-like eating in early-life trauma mice

We next asked whether the inhibition of LH^Lepr ^→ vlPAG neurons normalizes the ELT-induced maladaptive binge-like eating habits and vulnerability to obesity. To test this, we injected HSV-DIO-Flp into the vlPAG and AAV-fDIO-hM4Di-mCherry or AAV-fDIO-mCherry into the LH of ELT Lepr-Cre mice to ensure the expression of hM4Di receptors within LH^Lepr ^→ vlPAG neurons. Two weeks after surgery, we subjected these mice to repeated cycles of Re-HFD access with accompanying CNO administrations (2 mg per kg body weight, i.p.; Fig. 5g). Although ELT Lepr-Cre mice expressing mCherry alone showed the expected increase in binge-like eating over repeated cycles of Re-HFD, ELT Lepr-Cre mice with CNO-mediated inhibition of LH^Lepr ^→ vlPAG neurons showed normalization of the exacerbated binge-like eating phenotype (Fig. 5h). These data indicate that the inhibition of LH^Lepr ^→ vlPAG neurons can prevent the augmented and sustained binge-like eating habits associated with ELT.

Furthermore, to determine whether long-term suppression of LH^Lepr ^→ vlPAG neuronal activity rescues the tendency of ELT mice to develop HFD-induced obesity, we injected HSV-DIO-Flp into the vlPAG and AAV expressing the Flp-dependent Kir2.1 potassium channel (AAV-fDIO-eGFP-Kir2.1)^46^ into the LH of ELT Lepr-Cre mice, thereby inducing chronic hyperpolarization in the LH^Lepr ^→ vlPAG neurons (Fig. 5i). Indeed, chronic silencing of LH^Lepr ^→ vlPAG neurons significantly blocked the rapid weight gain observed in ELT mice expressing eGFP alone, while the same inhibition of the neurons was less likely to affect regular body weight gain in control mice (Fig. 5j). Together, these data strongly suggest that inhibition of LH^Lepr ^→ vlPAG neurons can alleviate the binge-like eating habits and obesity-prone characteristics induced by ELT.

### vlPAG^Penk^ is a functional downstream target of LH^Lepr^ neurons

Although the vlPAG contains various neurons that express diverse neuropeptides and neurotransmitters^47^, the molecular identity of the vlPAG that receives LH^Lepr^ neuronal inputs has not yet been described. The vlPAG neurons largely express the endogenous opioid peptide Penk, which is known to play diverse roles in regulating pain sensation, food intake and reward processing^48,49^. Given that several clinical studies have reported changes in endogenous opioidergic systems with BED and obesity^50^, we speculated that Penk-expressing vlPAG (vlPAG^Penk^) neurons may be the downstream target through which LH^Lepr^ neurons direct binge-like eating.

**Fig. 6:**
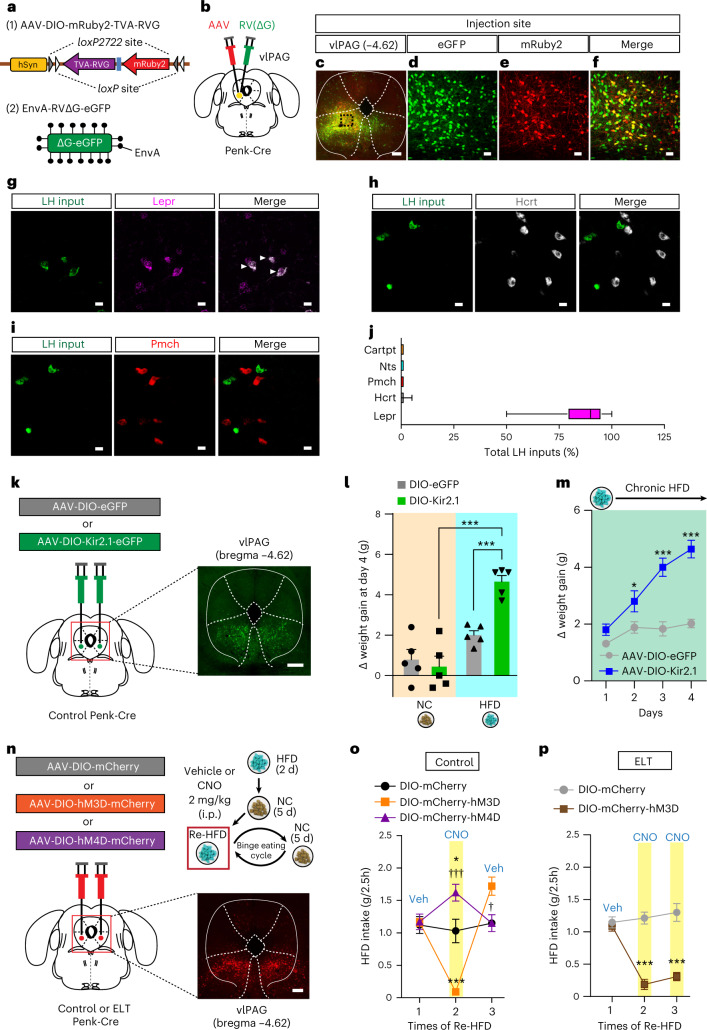
Activation of vlPAG^Penk^ neurons rescues binge-like eating habits of ELT mice. **a**,**b**, Schematics showing the strategy for rabies-mediated retrograde tracing of monosynaptic inputs to the vlPAG^Penk^ neurons. **c**–**f**, Confocal image showing starter cells in the vlPAG^Penk^ neurons (**c**); scale bar, 250 μm. Green, expressing eGFP (**d**), red, expressing mRuby2 (**e**), yellow, expressing both eGFP and mRuby2 (**f**), replicated independently with similar results in 3 mice. Scale bars, 50 μm. **g**–**i**, Representative images of LH neurons sending inputs to vlPAG^Penk^ neurons with mRNA labeling for Lepr (**g**), Hcrt (**h**) and Pmch (**i**), replicated independently with similar results in 2 mice. White arrows represent colocalization. Scale bars, 20 μm. **j**, Quantification of LH inputs expressing Lepr, Hcrt, Pmch, Nts and Cartpt to vlPAG^Penk^ neurons. Box plots display the median (center) and the 2.5th to 97.5th percentiles of the distribution (bounds) with whiskers extending from minimum to maximum values (*n* = 284 cells from two Penk-Cre mice). **k**, Schematic depicting the injection of an AAV expressing a Cre-dependent Kir2.1 into the vlPAG of control Penk-Cre mice, replicated independently with similar results in 5 mice. Scale bar, 500 μm. **l**,**m**, Cumulative body weight gain during 4 d of ad libitum access to either NC or HFD (*n* = 5 mice per group). In **l**, two-way ANOVA (*F*_(1,16) _= 13.580, *P* = 0.002) was followed by Fisher’s LSD post hoc test for multiple comparisons; ***P < 0.001 compared with HFD-fed mice expressing DIO-eGFP or NC-fed mice expressing DIO-Kir2.1; in **m**, two-way RM ANOVA (*F*_(3,24) _= 41.954, *P* < 0.001) was followed by Bonferroni post hoc test for multiple comparisons; *P = 0.02 and ***P < 0.001, compared with DIO-eGFP at the respective day. **n**, Schematic for the chemogenetic manipulation of vlPAG^Penk^ neurons. Confocal image showing DREADDs-expressing vlPAG^Penk^, replicated independently with similar results in 5 mice. Scale bar, 250 μm. **o**, 2.5 h Re-HFD consumption in control mice with or without CNO (*n* = 4, 6 and 5 mice for each group). Two-way RM ANOVA (*F*_(6,20) _= 22.438, *P* < 0.001) was followed by Bonferroni post hoc test for multiple comparisons; *P = 0.011 for mCherry versus hM4D; ***P < 0.001 for mCherry versus hM3D; ^†††^*P* < 0.001 and ^†^*P* = 0.022 for hM3D versus hM4D at the respective Re-HFD cycles. **p**, 2.5 h Re-HFD consumption in ELT mice with or without CNO (*n* = 5, 6 mice per group). Two-way RM ANOVA (*F*_(2,18) _= 23.197, *P* < 0.001) was followed by Bonferroni post hoc test for multiple comparisons; ***P < 0.001 compared with DIO-mCherry at the respective Re-HFD cycles. Data are the mean ± s.e.m.

**Extended Data Fig. 9:**
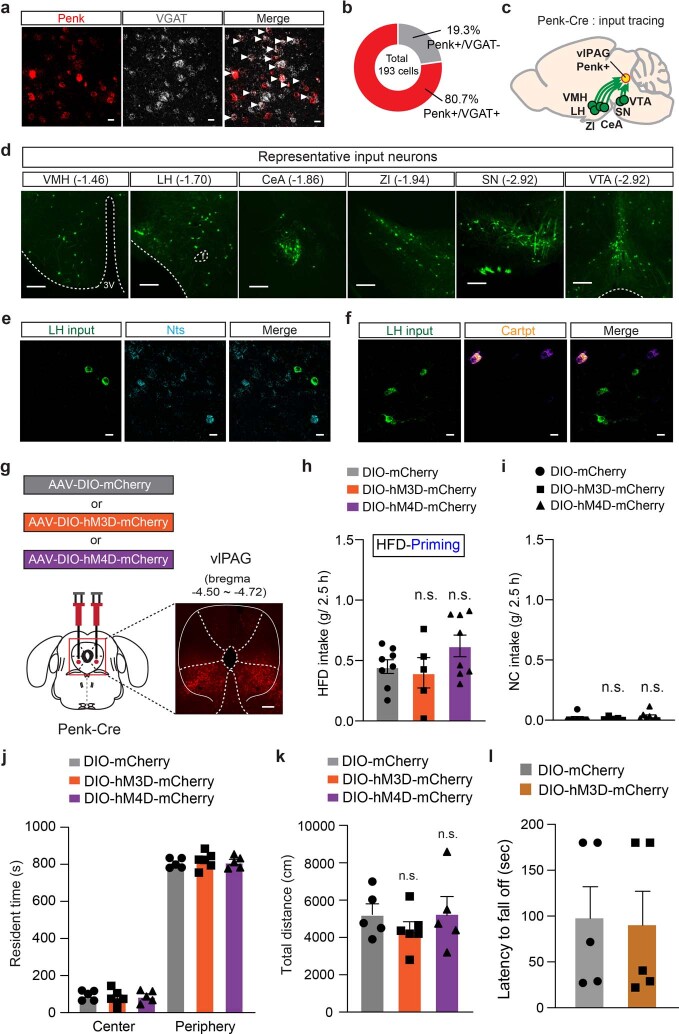
Effects of vlPAG^Penk^ neuronal modulation on food intakes and movements. (**a**) Representative images of Penk (red) and VGAT mRNA (white) in the vlPAG. White arrowheads show the colocalization, replicated independently with similar results in two mice. Scale bars, 20 μm. (**b**) Pie chart indicates percentage of Penk-positive neurons colocalizing with the probe to VGAT or not (n = 193 cells from two mice). (**c**) Schematic summary of the brain regions that provide inputs to the vlPAG^Penk^ neurons. (**d**) Images showing rabies-labeled presynaptic neurons in the VMH, LH, central amygdala (CeA), zona incerta (ZI), substantia nigra (SN), and VTA, replicated independently with similar results in three mice. Scale bars, 200 μm. (**e, f**) Representative images of LH neurons sending inputs to vlPAG^Penk^ neurons with mRNA labeling for Nts (**e**) and Cartpt (**f**), replicated independently with similar results in two mice. Scale bars, 20 μm. (**g**) Schematic depicting the viral injection for chemogenetic manipulation of vlPAG^Penk^ neurons in control Penk-Cre mice, replicated independently with similar results in three mice. Scale bar, 500 μm. (**h, i**) CNO-induced activation or inhibition of vlPAG^Penk^ neurons changes neither HFD intake at priming stage (h; n = 8, 5 and 8 mice for each group; One-way ANOVA [F(2,18)= 1.803, p = 0.193]) nor NC consumption in basal states (i; n = 6, 5 and 8 mice for each group; One-way ANOVA [F(2,16)= 0.619, p = 0.551]). (**j, k**) Chemogenetic manipulation of vlPAG^Penk^ neurons affects neither locomotor activity (**j**) nor the resident time spent in the center and periphery area of the open field (**k**) (n = 5, 6 and 5 mice for each group). In j, one-way RM ANOVA (F(2,13)= 0.633, p = 0.547); in k, two-way RM ANOVA (F(2,13)= 2.103, p = 0.162). (**l**) Latency to fall off during rotarod performance. Chemogenetic activation of vlPAG^Penk^ neurons of ELT mice does not elicit defects in motor coordination or balance (n = 5 mice per group). Two-tailed unpaired t-test, t8 = 0.143, p = 0.890. n.s., not significant. Data are presented as mean ± SEM.

Using dual FISH, we found that vlPAG^Penk^ neurons are primarily GABAergic (Extended Data Fig. 9a,b). To better understand the afferent connections of vlPAG^Penk^ neurons throughout the brain, we injected an AAV expressing Cre-dependent mRuby2, TVA receptor and rabies virus glycoprotein (RVG; AAV-DIO-mRuby2^−^TVA-RVG) into the vlPAG of Penk-Cre mice. Two weeks later, we delivered EnvA-pseudotyped, glycoprotein-deleted rabies virus (EnvA-RVΔG-eGFP) into TVA-expressing vlPAG to map neurons sending monosynaptic inputs to vlPAG^Penk^ (Fig. 6a–f). We detected the eGFP-labeled neurons at the whole-brain level and found that vlPAG^Penk^ neurons receive monosynaptic inputs from the LH, VMH, central amygdala, the zona incerta, midbrain neurons residing in the substantia nigra and VTA (Extended Data Fig. 9c,d).

Because the LH to vlPAG connection is poorly studied, we combined virus-mediated input tracing with FISH to investigate the molecular identity of the LH neurons projecting to the vlPAG^Penk^. Among the major cell types in the LH, we found the most extensive colocalization of rabies-eGFP-positive LH neurons with Lepr rather than with Pmch, Hcrt, Nts or Cartpt (Fig. 6g–j and Extended Data Fig. 9e,f). This approach revealed that the predominant inputs from the LH to vlPAG^Penk^ are the LH^Lepr^ neurons, suggesting that the vlPAG^Penk^ is likely a critical downstream target that relays information from the LH^Lepr^ neurons and leads to binge-like eating and obesity.

This led us to hypothesize that the direct regulation of vlPAG^Penk^ neuronal activity may control HFD-induced weight gain. As LH^Lepr^ neurons are primarily GABAergic (Extended Data Fig. 5b) and chronic inhibition of LH^Lepr ^→ vlPAG neuronal activity of ELT mice normalizes the obesity-prone characteristics (Fig. 5j), we reasoned that inhibition of vlPAG^Penk^ neurons of control mice may recapitulate the enhanced weight gain of ELT mice. To induce chronic inhibition in the vlPAG^Penk^ neurons, we injected an AAV expressing Cre-dependent Kir2.1 (AAV-DIO-eGFP-Kir2.1) into the vlPAG of control Penk-Cre mice (Fig. 6k). Intriguingly, Kir2.1-induced silencing of vlPAG^Penk^ neurons increased the weight gain under chronic HFD but not NC (Fig. 6l,m). These data support that inhibition of vlPAG^Penk^ neuronal activity is critical for enhancing the tendency toward HFD-induced obesity associated with ELT.

This result prompted us to examine whether the modulation of vlPAG^Penk^ neuronal activity can alter binge-like consumption. To test this idea, we injected AAV-DIO-hM3Dq-mCherry, AAV-DIO-hM4Di-mCherry or AAV-DIO-mCherry into the vlPAG of control Penk-Cre mice and then subjected them to Re-HFD with or without CNO (Fig. 6n). After CNO administration (2 mg per kg body weight, i.p.) at the 2nd Re-HFD cycle, chemogenetic inhibition of vlPAG^Penk^ neurons enhanced binge-like HFD consumption, while the activation of those neurons reduced it (Fig. 6o). However, the same manipulation of vlPAG^Penk^ neuronal activity did not change general HFD consumption during the priming stage, NC intake, locomotion or anxiety level (Extended Data Fig. 9g–k). Together, it is likely that manipulation of vlPAG^Penk^ neurons selectively alters the binge-like HFD eating in response to Re-HFD.

We then reasoned that activation of vlPAG^Penk^ neurons may normalize maladaptive binge-like eating habits of ELT mice. Indeed, CNO-mediated vlPAG^Penk^ neuronal activation prevented ELT mice from showing augmented and sustained binge-like eating upon multiple Re-HFD exposures with no accompanying changes in the motor coordination or regular movements (Fig. 6p and Extended Data Fig. 9l). Together, these data confirm that the activation of vlPAG^Penk^, as a primary downstream target of the LH^Lepr^ neurons, is required for rescue of maladaptive binge-like eating habits in ELT mice.

## Discussion

Here, we have delineated a new pathway in mice—through LH^Lepr^ neuronal projections to the vlPAG—that is a critical component for the binge-like eating habits and HFD-induced obesity associated with ELT in mice. Our observations demonstrate that ELT impairs Lepr signaling in the LH and that this contributes to increasing LH^Lepr^ neural activity that is associated with sustained binge-like eating. We have also shown that activation of LH^Lepr^ neurons projecting to the vlPAG play distinct roles in mediating the binge-like eating upon Re-HFD, whereas inhibition of the same neurons normalizes aberrant eating behaviors of ELT mice. Furthermore, direct activation of vlPAG^Penk^, which is a critical downstream target of LH^Lepr^ neurons, can rescue maladaptive eating habits of ELT mice.

In this study, we focused on the new finding that adult mice exposed to ELT exhibit augmented and sustained binge-like HFD consumption and vulnerability to HFD-induced obesity (Fig. 1). Notably, the current ELT paradigm was conducted in 3-day-old mouse pups that approximate the developmental stages of infancy in humans^51^. Given several human studies showing that child maltreatment under 18 years of age is related to psychopathology in adulthood^4,52^, future extended interrogation into the effects of ELT beyond P3 on maladaptive eating habits will be required.

At P4, ELT significantly decreased and increased levels of leptin and corticosterone, respectively. ELT may disrupt the so-called ‘stress hypo-responsive period’ during which mouse pups normally exhibit low basal corticosterone levels^53^, and thereby promote HPA axis activity and corticosterone levels. Stressful conditions that increase HPA axis activity influence appetite-related hormonal systems, such as leptin^54^. For example, chronic unpredictable mild stress induces depression-like behaviors in adult rats, accompanied by HPA axis hyperactivity and reduced hypothalamic Lepr mRNA levels, although the specific hypothalamic subregion was not clarified^55^. Future studies examining the interplay between HPA axis activity and the leptin system during early development will extend our understanding of ELT-induced long-term negative consequences such as pathological binge eating.

Activation of Lepr initiates multiple signal transduction pathways and modulates neuronal function by affecting ion channel activity or glutamate receptor trafficking. For example, Lepr activation in hippocampal neurons leads to JAK2/PI3K pathway activation that facilitates α-amino-3-hydroxy-5-methyl-4-isoxazolepropionic acid receptor (AMPAR) internalization^56^. This, in turn, would inhibit AMPAR-mediated synaptic transmission, thereby reducing neuronal activity. Given our data showing that Lepr knockdown increases the E/I ratio in LH^Lepr^ neurons (Fig. 2), we speculate that downregulation of Lepr signaling in the LH may trigger AMPAR insertion to the membrane and enhance glutamatergic synaptic transmission. Moreover, Lepr signaling modulates voltage-dependent K_v2.1_ or K_ATP_ channel activity, which plays a role in regulating intrinsic neuronal excitability^38,57^. Indeed, the excitability of LH^Lepr^ neurons was also increased in control mice expressing Lepr shRNA and ELT mice (Extended Data Fig. 5). Although further studies are needed to clarify the mechanisms by which Lepr signaling alters synaptic efficacy and LH neuronal excitability, our data provide important insight into the synaptic and cellular adaptations that underlie ELT or Lepr knockdown-induced binge-like eating habits.

We observed a large proportion of LH^Lepr^ neurons responding to eating bouts at the 1st Re-HFD, whereas a relatively small portion of the cells increases the activity during the HFD-priming or 4th Re-HFD stage (Extended Data Fig. 6). Given that the degree of desire for the HFD can be diminished after multiple repetitions or less likely to be developed at the initial trial, we speculated that LH^Lepr^ neuronal activity is preferentially engaged in the ‘internal state’ of eagerness for the HFD, thereby displaying the distinctive activation during the 1st Re-HFD. Furthermore, given that the LH^Lepr^ neuronal population has heterogeneous molecular compositions, it would be important to test whether the distinct functional clusters of LH^Lepr^ neurons show diverse responses at the onset of binge-like eating. Due to the low density of Lepr-expressing neurons in the LH, we were not able to image enough cells (10–12 cells per brain) for clustering analysis. Investigating distinct activity patterns in LH^Lepr^ neurons, beyond up and down changes, may provide further insight into binge-like eating habits.

We also identified two mostly distinct LH^Lepr^ neuronal populations projecting to either the vlPAG or the MPA (Fig. 4). Although both populations exhibit collateralized axonal terminals to some degree, we consider that the LH^Lepr ^→ vlPAG neurons send collateral projections to a greater extent than the LH^Lepr ^→ MPA neurons, based on relative fiber density across the brain. However, given that the efficacy of viral techniques is limited because viruses rely on cell-type-specific molecules for uptake and transport^58^, we acknowledge that we are targeting a subset of neuronal populations largely projecting to different brain areas. Although future works with extended methods may further disentangle the anatomical and structural connectivity of the LH^Lepr ^→ vlPAG and LH^Lepr ^→ MPA neurons, our viral strategy successfully differentiates the downstream target-defined LH^Lepr^ neuronal populations.

Importantly, we found activation or inhibition of LH^Lepr ^→ vlPAG neurons can either aggravate or alleviate the binge-like HFD consumption, whereas similar manipulations of LH^Lepr ^→ MPA neurons do not (Fig. 4). Although LH^Lepr ^→ vlPAG neurons send collateralized axons to other target structures, including the VTA and DRI, we speculate that the vlPAG is the most critical downstream structure in the regulation of binge-like HFD consumption for two reasons. First, optogenetic studies (Fig. 5) suggest that increased activity of LH^Lepr ^→ vlPAG neurons, but not of LH^Lepr ^→ VTA neurons, is required for binge-like consumption. Second, labeling the presynaptic terminals of LH^Lepr^ neurons (Extended Data Fig. 7) indicated that the vlPAG is the major target of LH^Lepr^ neurons for functional regulation. To the best of our knowledge, these are the first evidence identifying projection-specific LH^Lepr^ circuit that drives context-specific binge-like eating.

We identified vlPAG^Penk^ neurons as a crucial population that receives direct input from LH^Lepr^ neurons (Fig. 6). Given the GABAergic nature of LH^Lepr^ neurons, this is consistent with our finding that increased LH^Lepr ^→ vlPAG neuronal activity aggravates binge-like eating, likely by inhibiting vlPAG^Penk^ neuronal activity. We predict that reducing GABAergic LH^Lepr^ neuronal activity would relieve the inhibition of vlPAG^Penk^ neurons and drive further increases in their activity, attenuating binge-like eating. Our results provide a mechanistic framework by which LH^Lepr ^→ vlPAG^Penk^ pathway dysfunction could lead to binge-like eating habits and obesity. Furthermore, this indicates that interactions between the central leptin system and the endogenous opioidergic system may mediate the effects of ELT on binge eating habits and vulnerability to obesity.

Taken together, our results identify maladaptations induced by ELT that point to circuit mechanisms underlying abnormal eating habits. This may provide improved therapeutic strategies for the treatment of eating disorders and obesity caused by traumatic childhood experiences.

## Methods

### Mice

All mice, including wild-type C57BL/6J, Lepr-Cre (stock no. 008320) and Penk-Cre (stock no. 025112) mice, were obtained from the Jackson Laboratory. To visualize Lepr-positive neurons, Lepr-Cre mice were crossed with Ai14 mice (stock no. 007908; tdTom reporter line; Jackson Laboratory). All transgenic mice for behavioral experiments were backcrossed to wild-type C57BL/6J mice for multiple generations. A similar number of both male and female mice (10–13 weeks old) were used for body weight and food intake measurements, behavioral experiments, immunohistochemistry, RT–qPCR, FISH and anatomical tracing. Adult male and female mice (10–13 weeks old) were used for ex vivo slice electrophysiology, metabolic cage analysis and glucose tolerance tests. We initially examine the behaviors of males and females separately, but we didn’t find any significant difference. Mice were housed on a 12-h light/dark cycle with standard bedding in a temperature-controlled and humidity-controlled room (~21 °C and 42% humidity). All behavioral procedures were performed during the light cycle.

Experimenters were blind to the group allocation and outcome assessment. For data analysis, primary experimenters were not blind due because the experimental conditions (for example, stress paradigm, food exposure) were obvious to the researchers, but the analysis was carried out without subjective bias. All experiments were approved by the Institutional Animal Care and Use Committee of the Virginia Polytechnic Institute and State University and the University of California, San Diego.

### Early-life trauma procedures

The ELT paradigm was adopted from previously published methods with minor modifications^59,60^. Briefly, pregnant female mice were individually housed when they were 14–16 d pregnant. From P3 to P4, ELT pups were separated from both their dam and littermates for 23 h. During this separation, ELT pups were placed individually in a divided small chamber with clean bedding and transferred to an incubator, which was maintained at 32 ± 1 °C. At the end of the separation period, ELT pups were reunited with the dam and littermates. Control pups remained undisturbed in the maternal nest. All pups were weaned at P21 and housed in groups of three to five of the same gender until the start of the experiments.

### Binge-like eating paradigm

The binge-like eating behavior in mice was monitored using the repetitive intermittent-access schedule of a HFD (60% kcal% fat; D12492, Research Diets) with minor modifications of the previous publication^30^. The mice were fed standard NC (18% kcal% fat; no. 2918, Harlan-Teklad) until training and assessment of binge-like eating. Mice (10–13 weeks old) were singly housed and subjected to an initial 2 d of free access to HFD, then only NC was available ad libitum for the following 5 d. On the next day, mice were exposed to a HFD again (Re-HFD) and the food intake level was monitored 2.5 h later. After 24 h, the HFD was removed and replaced with NC only, thus completing the 1st binge eating cycle of Re-HFD. For repetitive cycles, mice were subjected to a 6-d NC-only period, which was followed by Re-HFD for 24 h. Foods were always available ad libitum with no food restrictions.

### Body weight and food intake measurements

Body weight changes or food intake of mice (10–13 weeks old) were recorded in their home cages for 3–4 weeks or 5 d of access to either NC or HFD. Food intake was assessed by subtracting the amount of food remaining in cages from the weight of foods initially provided.

### Metabolic phenotyping experiments

Mice were fed with a HFD and subjected to metabolic cage analysis to evaluate energy expenditure. Metabolic parameters including O_2_ consumption, CO_2_ production and respiratory exchange ratio were recorded by Comprehensive Lab Animal Monitoring System (Columbus Instruments)^61^ for three consecutive days and nights, with at least 24 h of an adaptation period before data recording.

### Serum leptin and corticosterone measurement

Mice were anesthetized with isoflurane and decapitated. Whole trunk blood samples were collected in 1.5 ml microcentrifuge tubes and allowed to clot at room temperature for 30 min. Tubes were then spun for 15 min at 3,000*g* at 4 °C. Supernatant containing clear serum was stored at −80 °C until enzyme-linked immunosorbent assay to measure serum leptin and corticosterone using an assay kit (EMD Millipore EZML-82K, and Cayman 501320, respectively).

### Glucose tolerance test

Glucose tolerance tests were done after mice underwent a fasting period of 16 h with water ad libitum. Mice were subsequently given i.p. injections of 100 mg ml^−1^ d-glucose (2 g per kg body weight). Five microliters of blood collected from the tail vein was dropped onto a glucose test strip (Breeze 2 blood glucose meter kit, Bayer). Blood glucose was determined at 0, 40 and 120 min after the injection.

### Locomotion

Locomotion was assessed in an open field arena (44 × 44 × 44 cm^3^). Mice were placed individually in the arena and allowed to explore freely for 15 min. The activity was monitored using a webcam mounted above the arena and analyzed by tracking software (Viewer 3.0, BIOBSERVE).

### Open field test

Each mouse was placed in the open field arena (44 × 44 × 44 cm^3^) and allowed to move freely for 15 min. The open field area was subdivided into two zones, a center (20 × 20 cm^2^) and a periphery. For the chemogenetic experiment, CNO (2 mg per kg body weight, i.p.) was administered 30 min before the open field test session. The movement of mice was monitored with a webcam and analyzed by tracking software (Viewer 3.0, BIOBSERVE).

### Elevated plus maze

The elevated plus maze consisted of two open arms, two closed arms and a center elevated to a height of 30.5 cm above the ground. Mice were placed in the center and allowed to explore the space for 5 min. For the chemogenetic experiment, CNO (2 mg per kg body weight, i.p.) was administered 30 min before the elevated plus maze session. The movement of mice was analyzed by tracking software (Viewer 3.0, BIOBSERVE).

### Novel object recognition test

Mice were habituated to the open field arena (44 × 44 × 44 cm^3^) in the absence of objects for 30 min a day before the training session. During the training session, two identical objects were placed in each corner of the arena, and mice were allowed to explore for 10 min. Twenty-four hours after training, mice were placed in the arena where one of the two objects was replaced with a novel object having a different color and shape. All movements of mice were monitored with a webcam for 10 min, and the recognition time in each object area (2 cm around the object) was measured by tracking software (Any-maze). Discrimination rate was calculated as ((time spent in a novel object area) / (time spent in a novel object area + time spent in a familiar object area) × 100(%))^42,62^.

### Rotarod test

For the fixed speed rotarod test (17 r.p.m.), each mouse was given two practice trials and then placed on the rotating cylinder in a rotarod apparatus (Ugo Basile). The latency to fall off the rotarod was recorded with a 3-min cutoff per session^63^.

### Immunohistochemistry

For c-fos immunoreactivity, mice were individually housed before the training and assessment of binge-like eating behavior. After the HFD withdrawal period (5 d), a HFD was reintroduced to mice for 2.5 h. Subsequently, the mice were then anesthetized with isoflurane and transcardially perfused with 4% paraformaldehyde (PFA). Brains were extracted and post-fixed overnight in 4% PFA. Coronal sections (50 μm) were washed in PBS containing 0.3% Triton X-100 (PBS-T, pH 7.4) and incubated in 1% BSA (vol/vol) in PBS-T for 1 h. The sections were immunostained using a rabbit anti-c-fos antibody (1:5,000 dilution; Cell Signaling Technology) applied overnight in PBS-T, at room temperature. On the next day, sections were washed in PBS-T and incubated in a 1:500 dilution of Alexa Fluor Plus 488 anti-rabbit secondary antibody (Thermo Fisher Scientific) in PBS-T for 1 h. For Mch and Hcrt immunostaining, rabbit anti-Mch (1:1,000 dilution; Phoenix Pharmaceuticals) and anti-Hcrt (1:1,000 dilution; Phoenix Pharmaceuticals) were applied. For phospho-STAT3 (pSTAT3) immunofluorescence staining, rabbit anti-pSTAT3 antibodies (1:500 dilution; Cell Signaling) were applied overnight at room temperature. Thereafter, the sections were rinsed with PBS-T and incubated for 1 h with horseradish peroxidase-conjugated anti-rabbit secondary antibody (1:1,000 dilution; Cell signaling Technology) in PBS-T. Subsequently, sections were washed and incubated for 10 min with a 1:50 dilution of tyramide signal amplification (TSA) Plus Fluorescein (TSA Plus kit, PerkinElmer Life Sciences) to enhance the pSTAT3 signal. Sections were rinsed with PBS-T and mounted using a mounting medium. Images were acquired using a confocal microscope (Olympus FluoView FV1200) and quantitatively analyzed with ImageJ. For c-fos and pSTAT3 quantification, the neurons located lateral and up to 0.1 mm medial to the fornix or up to 0.2 mm above or below the fornix were considered to be in the LH. Cell counts were made within this reference area by applying equal thresholds to all images and using the ‘analyze particles’ function in ImageJ. For c-fos quantification in Lepr-Cre × Ai14 mice, c-fos and tdTom double-positive cells were counted. The percentage of double-positive cells was calculated among the total numbers of tdTom-expressing cells.

### RT–qPCR

Mice were anesthetized with isoflurane and 250-µm slices were prepared in PBS, using a vibratome (Leica VT1200). The LH was microdissected bilaterally, and samples were immediately frozen on dry ice and stored at −80 °C before RNA isolation. Total RNA was extracted from dissected samples using a Hybrid-R RNA purification kit (GeneAll Biotechnology). Purified RNA samples were reverse transcribed by using the SuperScript-IV First-strand cDNA synthesis kit (Invitrogen). qPCR was performed by using TaqMan Gene Expression Assay Kit (Applied Biosystems). All TaqMan probes were purchased from Applied Biosystems and are as follows: Lepr (Mm00440181_m1), Hcrtr1 (Mm01185776_m1), Mchr1 (Mm00653044_m1), Hcrt (Mm01964030_s1), Pmch (Mm01242886_g1), Galr1 (Mm00433515_m1), Nts (Mm00481140_m1), Gal (Mm00439056_m1), Cartpt (Mm04210469_m1) and glyceraldehyde-3-phosphate dehydrogenase (GAPDH; Mm99999915_g1). Target amplification was performed by using ViiA 7 Real-Time PCR System (Applied Biosystems) with QuantStudio Real-Time PCR software v1.3. The relative mRNA expression levels were calculated via a comparative threshold cycle (C_t_) method using GAPDH as an internal control: ΔC_t_ = C_t_ (gene of interest) − C_t_ (GAPDH). The gene expression fold change, normalized to the GAPDH and relative to the control sample, was calculated as the 2^−ΔΔCt^ methods^64^.

### RNA in situ hybridization

Brains were rapidly extracted and flash frozen with isopentane (Sigma-Aldrich) chilled with dry ice in 70% ethanol. Coronal brain slices (16 µm) containing the LH were sectioned using a cryostat (Leica CM3050S) at −20 °C. Brain slices were mounted directly onto slides and stored at −80 °C until RNA in situ hybridization which was conducted using RNAscope probes (Advanced Cell Diagnostics, ACD). Slides were fixed in 4% PFA for 15 min at 4 °C and subsequently dehydrated for 5 min with 50%, 70% and 100% ethanol at room temperature. Sections were then incubated with a Protease IV solution for 30 min, and washed with PBS, before being incubated with probes for 2 h at 40 °C in the HybEZ oven (ACD). All probes used were commercially available: Mm-Lepr (402731), Mm-Hcrt (490461), Mm-Pmch (478721), Cre (312281), Mm-Cartpt (432001), Mm-Nts (420441) and Mm-Slc32a1 (319198). After washing with wash buffer, the signal was amplified by incubating tissue sections in amplification buffers at 40 °C. After the final rinse, DAPI solution was applied to the sections. Slides were visualized with a confocal microscope (Olympus FluoView FV1200).

### Circuit mapping quantification

For LH^Lepr^ neuronal downstream connectivity quantitation, eGFP-labeled fibers and synaptic puncta were quantified as previously described with slight alterations^65,66^. Briefly, 50-µm coronal sections were obtained across the anteroposterior axis of the brain. Brain regions were determined by anatomical landmarks and based on the Paxinos and Franklin mouse brain atlas^67^. Images were taken with an Olympus FluoView FV1200 confocal microscope. Analysis was conducted in Fiji (ImageJ) and quantified as a percentage of the area of thresholded pixels normalized to either the HSV-DIO-Flp injection sites (Fig. 4c,f) or the vlPAG (Extended Data Fig. 7n,o). Qualitatively determined threshold values were obtained by determining the level that best mirrored the original image without introducing a background and maintained the consistency throughout animals.

### Virus and shRNA generation

AAV was produced by transfection of 293 cells with three plasmids: an AAV vector expressing target constructs (EmGFP, EmGFP-Lepr shRNA, DIO-EmGFP, DIO-EmGFP-Lepr shRNA, saCas9, Lepr sgRNA (a gift from D. Kong), DIO-GCaMP6f, DIO-eGFP, DIO-Synaptophysin-eGFP, DIO-eNpHR3.0-eYFP, DIO-eYFP, fDIO-mCherry, fDIO-hM3D-mCherry, fDIO-hM4D-mCherry, fDIO-eGFP, fDIO-Kir2.1-eGFP, DIO-mCherry, DIO-mCherry-hM3D, DIO-mCherry-hM4D and DIO-mRuby2-TVA-RVG), AAV helper plasmid (pHELPER; Agilent) and AAV rep-cap helper plasmid (pRC-DJ, a gift from M. Kay). At 72 h after transfection, the cells were collected and lysed. Viral particles were then purified by an iodixanol step-gradient ultracentrifugation method. The iodixanol was diluted and the AAV was concentrated using a 100-kDa-molecular-mass-cutoff ultrafiltration device. The genomic titer was determined by qPCR. The AAV vectors were diluted in PBS to a working concentration of approximately 10^12^ viral particles per ml. To generate EnvA-pseudotyped glycoprotein (G)-deleted rabies virus expressing eGFP (RVΔG-eGFP), we followed a published protocol^68^. Plasmids expressing the rabies viral components, B7GG, BHK-EnvA and HEK-TVA cells were provided courtesy of E. M. Callaway. HSV-DIO-Flp was purchased from the Gene Delivery Technology Core at the Massachusetts General Hospital.

To construct shRNA against Lepr (NM_146146.3), oligonucleotides that contained 21-base-pair sense and antisense sequences (5′-AACTGATGAAGAGCAAGGGTT-3′) targeting Lepr were connected with a hairpin loop followed by a poly(T) termination signal. This shRNA oligonucleotide was ligated into BLOCK-iT POLII miR RNA-mediated interference expression vectors (Invitrogen) and then transferred to an AAV vector together with EmGFP. To test the efficacy of the shRNA, we stereotaxically injected AAVs expressing Lepr shRNA into the LH. Two weeks after injection, the virus-infected area labeled by EmGFP expression was dissected. Lepr mRNA levels were measured by qPCR and found to be reduced over 70% (Fig. 2d and Extended Data Fig. 3l).

### Stereotaxic surgeries and histology

Lepr-Cre or wild-type mice (8–10 weeks old) were anesthetized with a mixture of ketamine (100 mg per kg body weight) and dexmedetomidine (0.5 mg per kg body weight). The mouse was mounted in a stereotaxic frame (David Kopf Instruments). Body temperature was kept stable by using a heating pad while recovering from anesthesia. Viral injections were targeted using coordinates based on the Paxinos and Franklin mouse brain atlas^67^.

For shRNA-related testing in behavior or electrophysiology experiments, viral preparations (AAV-EmGFP, AAV-EmGFP-Lepr shRNA, AAV-DIO-EmGFP, AAV-DIO-EmGFP-Lepr shRNA) in a 300–350-nl volume were injected bilaterally into the LH (bregma, anteroposterior −1.65 mm; lateral ±1.12 mm; dorsoventral −5.25 mm) at a slow rate (100 nl min^−1^) using a syringe pump. Mice were allowed 2 weeks to recover from the virus injections before starting of either behavioral tests or electrophysiological recording. Injection sites were confirmed in all animals by preparing coronal sections containing the desired plane, and animals with incorrect injection placement were excluded from analyses.

For the CRISPR–SaCas9 viral-based system, both the AAV carrying an sgRNA targeting the mouse Lepr locus (AAV-sgLepr^38^, gift from D. Kong) and the AAV expressing *S. aureus* Cas9 (SaCas9; AAV-hSyn-SaCas9-U6-sgRNA) were bilaterally co-injected into the LH of wild-type mice. Three weeks were given for viral expression before behavioral tests.

For in vivo Ca^2+^ imaging experiments, Lepr-Cre animals received a unilateral injection of AAV-DIO-GCaMP6f (250 nl) into the LH. After 10–15 min of viral infusion, a sterile 20-gauge needle was slowly lowered into the same site to a depth of −4.65 mm from the cortical surface to clear a path for the implantation of a GRIN lens. The snap-in imaging cannula (model L-V; 500 µm in diameter; 5.66 mm in length; Doric Lenses) with GRIN lens was then implanted above the virus injection site. The target depth of the lens was adjusted to 100 µm above the viral injection site. The implanted imaging cannula and focusing ring were secured to the skull with an initial layer of adhesive cement (C&B Metabond; Parkell) followed by a second layer of dental cement (Ortho-Jet; Lang). In vivo Ca^2+^ imaging tests were performed 6 weeks after the implantation of the image cannula.

For anatomical output mapping, either AAV-DIO-eGFP or AAV-DIO-synaptophysin-eGFP (300 nl) were unilaterally injected into the LH of Lepr-Cre mice. Mice were euthanized 2 weeks after the viral injection for examining the outputs of LH^Lepr^ neurons. For input mapping experiments with EnvA-pseudotyped rabies virus, we first unilaterally injected 300 nl of AAV-DIO-mRuby2-TVA-RVG into the vlPAG (bregma, anteroposterior −4.62 mm; lateral ±0.3 mm; dorsoventral −2.82 mm) of Penk-Cre mice. Two weeks later, mice were again anesthetized as previously described and injected with EnvA-pseudotyped RVΔG-eGFP into the same site, vlPAG. The mice were euthanized 5 d after the last injection for input mapping analysis.

For optogenetic behavioral experiments, Lepr-Cre animals were bilaterally injected with 350 nl of AAV-DIO-eYFP or AAV-DIO-eNpHR3.0-eYFP into the LH. Bilateral chronic optic fibers (200 µm in diameter, 0.22 NA; Doric Lenses) were implanted above the downstream targets of LH^Lepr^ neurons in either vlPAG or VTA (bregma, anteroposterior −3.15 mm; lateral ±0.4 mm; dorsoventral −4.42 mm). Implanted fibers were adhered to the skull with adhesive and dental cement as described above. Lastly, sutures or sterile tissue adhesive (Vetbond; 3M) was used to close the incision. Three weeks were given for viral expression in terminals before behavioral tests. Upon completion of the behavioral experiment, viral injections and fiber placement were confirmed.

For the manipulation of LH^Lepr^ neurons in a projection-specific manner, Lepr-Cre animals were bilaterally injected with 350 nl of AAV-fDIO-mCherry, AAV-fDIO-mCherry-hM3D, AAV-fDIO-mCherry-hM4D, AAV-fDIO-eGFP or AAV-fDIO-Kir2.1-eGFP into the LH. During the same surgery session, HSV-DIO-Flp (350 nl) was injected into either vlPAG or MPA (bregma, anteroposterior −0.11 mm; lateral ±0.25 mm; dorsoventral −5.35 mm). For manipulation of vlPAG^Penk^ neurons, AAV-DIO-mCherry, AAV-DIO-mCherry-hM3D, AAV-DIO-mCherry-hM4D, AAV-DIO-eGFP or AAV-DIO-Kir2.1 was bilaterally injected (300 nl) into the vlPAG of Penk-Cre mice. Three weeks were given before behavioral testing.

For microinjection of the leptin or PESLAN into the LH, a guide cannula (26-gauge, 6-mm long; Plastics One) was chronically implanted in this brain region (bregma, anteroposterior −1.65 mm; lateral ±1.12 mm; dorsoventral −5.05 mm). Implanted cannulae were secured to the skull as described above, and then obturators were placed in the guide cannulae. Behavioral testing was performed 2 weeks after the implantation. Upon completion of all behavioral experiments, viral injections or fiber/cannula placements were confirmed. Mice with off-target expression and/or off-target implant tip location were excluded from the final analyses.

After each experiment, the extent of viral transduction spread was examined at the conclusion and viral transduction was validated whether it was limited to the LH without off-target effects in other adjacent areas such as the Arc, VMH and DMH. The inclusion criteria were applied when the virally labeled neurons were located in the LH reference area; that is, lateral and up to 0.1 mm medial to the fornix or up to 0.2 mm above or below the fornix. If the viral expression was found outside this reference area or the viral transduction was weak in the LH (covering less than 50% of the total LH area), the mice were excluded from the final dataset, which was determined by two experimenters who were blinded to the experimental design.

### Optogenetic stimulation

A 3-m-long fiber-optic patch cord (Doric Lenses) was connected to the chronically implanted optic fiber and suspended above the behavioral testing area to allow animals to move freely while receiving laser illumination. For eNpHR3.0-mediated inhibition, the patch cord was connected to a 593-nm laser (OEM Laser Systems). Bilateral inhibition of the LH^Lepr ^→ vlPAG or LH^Lepr ^→ VTA circuit was achieved by delivering continuous yellow light for 30 min. The power of the light at the tip of each optic fiber was adjusted to 10 mW. On the day of testing, each mouse was then placed in an experimental chamber (22 × 22 × 22 cm^3^) with a transparent acrylic bottom, which is located 40 cm above the webcam. After a 30 min habituation to the chamber and the patch cord, either HFD or Re-HFD was introduced to the mouse with optical stimulation (ON) for 30 min. All behaviors were recorded with a bottom view^69^ to analyze the latency to the first eating bout, eating duration, and physical contact to the food.

### In vivo Ca^2+^ imaging in freely moving mice and data analysis

For in vivo Ca^2+^ imaging experiments, mice were exposed to HFD priming or Re-HFD. On the testing day, mice were habituated to a clear glass chamber (25 cm in height, 20 cm in diameter) for 30 min with the head-mounted microscope body attached to the top of the imaging cannula. To monitor the GCaMP6f fluorescence change during binge-like eating behaviors, Re-HFD was presented for 2 min with a previous recording of baseline fluorescence for the same period of time. Mice eating behaviors were recorded by a web camera concurrently. Images were acquired at 8 frames per second with an average exposure time of 125 ms using Doric Neuroscience Studio software (version 5.3.1.2; Doric Lenses). LED power was maintained at 30% with analog gain 2. All image analysis with Δ*F/F*_0_ was performed with a Doric Neuroscience Studio software (Doric Lenses). To remove movement artifacts, individual image frames were aligned using a single frame as a reference, and then background fluorescence was removed from the aligned images. Regions of interest corresponding to cell bodies were determined using an automated cell-finding function and were visually inspected to ensure accuracy. Neural Ca^2+^ dynamics (Δ*F/F*_0_) were presented as a heat map using customized MATLAB code. The Δ*F/F*_0_ values were smoothed with a Gaussian filter and sorted in descending order after the introduction of Re-HFD. For the classification of neurons, the basal level of Δ*F/F*_0_ was determined for 2 min before the food presentation. Cells were considered as up or down if the Δ*F/F*_0_ value during the first 2 min after presenting Re-HFD was higher than basal Δ*F/F*_0_ + standard deviation (*σ*) or lower than basal Δ*F/F*_0_ − *σ*, respectively. Cells with average fluorescence between basal Δ*F/F*_0_ + *σ* and Δ*F/F*_0 _− σ were categorized as non-response. To detect individual Ca^2+^ transients, we first processed the Δ*F/F*_0_ time series to remove slow drifts, estimated by a median filter of 10 s in width. Next, the median absolute deviation (MAD) of the entire time series was computed. Ca^2+^ transients were extracted by sequentially detecting each upward transient that exceeded a 6-MAD threshold (equivalent to 4 *σ* assuming normal distribution) and following the previous event by an interval no shorter than 2 s. For correlating LH^Lepr^ neuronal activity with eating bouts, Ca^2+^ transients of cells from individual animals aligned to the first eating bout onset. For 1.5 min after the first eating bout, the number of cells producing at least one Ca^2+^ transient that occurred at the onset of eating bouts in the presence of HFD were counted. The percentage of the correlated or non-correlated cells was calculated among the total numbers of LH^Lepr^ neurons that were detected during the image session. It was challenging to get a large number of the LH^Lepr^ neurons per animal in this area for clustering analysis. In addition, because of the difficulty of tracking same neurons over a long period of time, our analysis focused on the changes within sessions.

For in vivo Ca^2+^ imaging experiments with either a novel object (for example, Lego brick) or a social stimulus (for example, mice urine from the opposite sex conspecifics), the individual mouse was habituated to a clear glass chamber (25 cm in height, 20 cm in diameter) for 30 min with the head-mounted microscope body attached to the top of the imaging cannula. The basal level of average cell fluorescence (Δ*F/F*_0_) was determined for 2 min before either novel object or mice urine application.

### Ex vivo electrophysiology

Coronal brain slices (300 µm) containing the LH were prepared using a vibratome (Leica VT1200S), in a solution containing: 110 mM choline chloride, 25 mM NaHCO_3_, 1.25 mM NaH_2_PO_4_, 2.5 mM KCl, 7 mM MgCl_2_, 25 mM glucose, 0.5 mM CaCl_2_, 11.6 mM ascorbic acid and 3.1 mM pyruvic acid, saturated with 95% O_2_/5% CO_2_. Slices were then allowed to recover at 30 °C for 20 min, and subsequently at room temperature, in a solution containing: 118 mM NaCl, 26 mM NaHCO_3_, 11 mM glucose, 15 mM HEPES, 2.5 mM KCl, 1.25 mM NaH_2_PO_4_, 2 mM pyruvic acid, 0.4 mM ascorbic acid, 2 mM CaCl_2_ and 1 mM MgCl_2_, saturated with 95% O_2_/5% CO_2_. Slices were maintained at room temperature for at least 1 h until transferred to a recording chamber perfused with: 119 mM NaCl, 26.2 mM NaHCO_3_, 11 mM glucose, 2.5 mM KCl, 1 mM NaH_2_PO_4_, 2.5mM CaCl_2_ and 1.3 mM MgCl_2_, saturated with 95% O_2_/5% CO_2_, and delivered at 2 ml min^−1^ at 30 ± 1 °C. For all recordings, patch pipettes (3–5 MΩ) were pulled from borosilicate glass (G150TF-4; Warner Instruments) with a DMZ Universal Electrode Puller (Zeitz Instruments) and filled with appropriate intracellular solutions. Neurons were visualized with differential interference contrast optics or epifluorescence (Olympus). Recordings were made with a MultiClamp 700B amplifier and pClamp10 software (Molecular Devices). Data were low-pass filtered at 1 kHz and digitized at 10 kHz with a digitizer (Digidata 1440; Molecular Devices). Series resistance was monitored and cells that displayed a change > 20% throughout recording were excluded.

To measure the E/I ratio using voltage-clamp recording, pipettes were filled with a Cs-based intracellular solution, containing: 115 mM Cs^+^-methanesulphonate, 10 mM HEPES, 1 mM EGTA, 1.5 mM MgCl_2_, 4 mM Mg^2+^-ATP, 0.3 mM Na^+^-GTP, 10 mM Na_2_-phosphocreatine, 2 mM QX 314-Cl and 10 mM BAPTA-tetracesium (295 mOsm, pH 7.35). Electrically evoked excitatory postsynaptic and inhibitory postsynaptic currents (eEPSCs and eIPSCs, respectively) were recorded from identified LH^Lepr^ neurons at −70 mV (for EPSCs) and 0 mV (for IPSCs). The E/I ratio was calculated by dividing the amplitude of eEPSCs by the amplitude of eIPSCs.

To measure the intrinsic excitability and rheobase using current-clamp recording, pipettes were filled with an intracellular solution containing: 125 mM K^+^-gluconate, 4 mM NaCl, 10 mM HEPES, 0.5 mM EGTA, 20 mM KCl, 4 mM Mg^2+^-ATP, 0.3 mM Na^+^-GTP and 10 mM Na_2_-phosphocreatine (290–300 mOsm, pH 7.2). To measure the firing capacity of LH^Lepr^ neurons, baseline firing was maintained and currents of increasing intensity (10-pA increments, duration of 500 ms) were injected up to 300 pA. Rheobase was determined as the minimal current step eliciting at least one action potential. All current-clamp recordings were performed in the presence of 5 µM NBQX (2,3-dihydroxy-6-nitro-7-sulfamoyl-benzo(F)quinoxaline) and 50 µM picrotoxin to block synaptic transmission. Analyses were performed offline using Clampfit (Molecular Devices).

### Intraperitoneal drug administration

For chemogenetic manipulations, CNO (Enzo Life Sciences) was dissolved in sterile distilled water. For the HFD intake experiments, mice were administered with CNO (2 mg per kg body weight, i.p.) or an equivalent volume of distilled water right before subjecting them to either HFD or Re-HFD. HFD intake was monitored for 2.5 h after CNO administration. For NC intake experiments, CNO (2 mg per kg body weight, i.p.) was administered either when NC was presented back after a 5-h food deprivation or during the basal state. Total NC intake levels were measured for 2.5 h after CNO administration. In the experiments analyzing pSTAT3 levels, leptin (Tocris Bioscience) was dissolved in sterile saline. Mice received an i.p. injection of leptin (1 mg per kg body weight) or saline 1 h before transcardial perfusion.

### Intracranial drug injection and histology

On the day of the intracranial injection, an internal cannula (33-gauge, projecting 0.2 mm below the tip of the guide; Plastics One) connected to 1-ml syringes (Hamilton) via polyethylene (PE)−20 tubing was inserted into the guide. Leptin (1 µg per 0.5 µl per side, Tocris Bioscience) or saline was microinjected bilaterally into the LH when the NC was presented back after 5 h of food deprivation. Body weight and NC intake were monitored for 24 h after leptin administration.

For pegylated superactive mouse leptin antagonist (PESLAN, p.Asp23Leu/p.Leu39Ala/p.Asp40Ala/p.Phe41Ala mutant; Protein Laboratories Rehovot)^70^ experiment, sterile distilled water or PESLAN (1 or 2.5 µg per 0.5 µl per side) was bilaterally microinjected into the LH once a day during chronic NC or HFD exposure. Body weight changes of mice were recorded in home cages for 5 d of access to either NC or HFD. A high dose of PESLAN (2.5 µg per 0.5 µl per side) was microinjected bilaterally into the LH 5 min before the Re-HFD exposure in each binge-like eating cycle. HFD intake level was measured for 2.5 h.

For the chemogenetic experiment, CNO (1 mM, dissolved in artificial cerebrospinal fluid, 0.5 µl per side) was microinfused bilaterally into the vlPAG 5 min before Re-HFD exposure and the HFD intake level was monitored for 2.5 h.

Upon completion of experiments, mice were anesthetized and perfused with 4% PFA. Brains were extracted and further post-fixed in 4% PFA. Coronal sections (50 µm) were subsequently stained with 0.2% cresyl violet (Sigma-Aldrich) for the verification of cannula tip placements. Only mice with injection cannula tips located bilaterally in the LH or vlPAG were included in the data analysis.

### Sample size and statistics

Samples sizes required for this study were initially estimated based on pilot studies, but no formal statistical tests were used to predetermine sample size. However, a power analysis was conducted to validate the sample size and the endpoint (significance level at 0.05 and power at 0.9).

If the viral expression was found outside this reference area or the viral transduction was weak in the LH (covering less than 50% of the total LH area), we excluded the mice from the final dataset, which was determined by two experimenters who were blinded to the experimental design. This exclusion happened in two wild-type mice (Extended Data Fig. 3n) due to the viral expression in VMH, one wild-type mouse (Extended Data Fig. 4a) due to off-target cannula implantation and one Lepr-Cre mouse (Fig. 5j) due to weak viral transduction.

We required the use of a group of animals with specific ages and a history of stress; however, within a group, we randomly chose animals for experiments. Animals used in this study were not selected based on any other prerequisite features other than general animal well-being (for example, normal grooming and social behavior, no obvious infections) for allocation into a particular experimental group.

Differences across more than two groups were analyzed with one-way or two-way ANOVA followed by Bonferroni or Fisher’s LSD post hoc tests for multiple comparisons. For comparisons between two groups, two-tailed *t*-tests (paired or unpaired) were used as described in the figure legends. The distribution of data was checked for normality and equal variance. *P* < 0.05 was considered statistically significant. Data are reported as the mean ± s.e.m. All statistical tests were performed using Prism version 8 for Windows, GraphPad Software (https://www.graphpad.com/).

### Reporting summary

Further information on research design is available in the Nature Portfolio Reporting Summary linked to this article.

## Online content

Any methods, additional references, Nature Portfolio reporting summaries, source data, extended data, supplementary information, acknowledgements, peer review information; details of author contributions and competing interests; and statements of data and code availability are available at 10.1038/s41593-022-01208-0.

## Supplementary information

Editorial Assessment Report

Reporting Summary

## Data Availability

Because of the size and complexity of the datasets, the data that support the findings of this study are available from the corresponding authors upon reasonable request.
