# Supplementary material for: Early adversity promotes binge-like eating habits by remodeling a leptin-responsive lateral hypothalamus–brainstem pathway
Source: Nat Neurosci. 2022 Dec 12;26(1):79–91. doi: 10.1038/s41593-022-01208-0 (PMC9829538; doi:10.1038/s41593-022-01208-0)
Supplement: Supplementary file 1 — Editorial Assessment Report [file 41593_2022_1208_MOESM1_ESM.pdf]

nature neuroscience

Article

https://doi.org/10.1038/s41593-022-01208-0

Early adversity promotes binge-like eating habits by remodeling a leptin-responsive lateral hypothalamus–brainstem pathway

In the format provided by the authors and unedited

Contents of this report

1. **Manuscript details**: overview of your manuscript and the editorial team.
2. **Review synthesis**: summary of the reviewer reports provided by the editors.
3. **Editorial evaluations**: personalized evaluation and recommendation from all 3 journals.
4. **Annotated reviewer comments**: the referee reports with comments from the editors.
5. **Open research evaluation**: advice for adhering to best reproducibility practices.

About the editorial process

Because you selected the **Nature Portfolio Guided Open Access** option, your manuscript was assessed for suitability in three of our titles publishing high-quality work in your field of research. More information about Guided Open Access can be found here.

Collaborative editorial assessment

Your editorial team discussed the manuscript to determine its suitability for the Nature Portfolio Guided OA pilot. Our assessment of your manuscript takes into account several factors, including whether the work meets the technical standard of the Nature Portfolio and whether the findings are of immediate significance to the readership of at least one of the participating journals in the Guided OA pilot.

Peer review

Experts were asked to evaluate the following aspects of your manuscript:

- **Novelty** in comparison to prior publications;
- **Likely audience** of researchers in terms of broad fields of study and size;
- **Potential impact** of the study on the immediate or wider research field;
- **Evidence** for the claims and whether additional experiments or analyses could feasibly strengthen the evidence;
- **Methodological detail** and whether the manuscript is reproducible as written;
- Appropriateness of the **literature review**.

Editorial evaluation of reviews

Your editorial team discussed the potential suitability of your manuscript for each of the participating journals. They then discussed the revisions necessary in order for the work to be published, keeping each journal's specific editorial criteria in mind.

Journals in the Nature portfolio will support authors wishing to transfer their reviews and (where reviewers agree) the reviewers' identities to journals outside of Springer Nature.
If you have any questions about review portability, please contact our editorial office at guidedoa@nature.com.

Manuscript details

Tracking number	Submission date	Decision date	Peer review type
GUIDEDOA-21-00309	Oct 27, 2021	Jan 6, 2022	Single-blind

Manuscript title

Early adversity promotes binge-like eating habits by remodeling a leptin-responsive circuit that connects the lateral hypothalamus to the brainstem

Author details

Dr Byung Kook Lim

Affiliation:
Neurobiology Section, Division of Biological Sciences Division, University of California San Diego

Editorial assessment team

**Primary editor**	**Leonie Welberg**<br>**Home journal: *Nature Neuroscience***<br>**ORCID:** orcid.org/0000-0001-7619-9149<br>**Email:** l.welberg@nature.com
**Other editors consulted**	**Noah Gray**<br>**Home journal: *Nature***<br>**ORCID:** https://orcid.org/0000-0002-4672-7403<br><br>**Christian Schnell**<br>**Home journal: *Nature Communications***<br>**ORCID:** https://orcid.org/0000-0002-3499-9217
**About your primary editor**	Leonie Welberg received her PhD from Edinburgh University and did postdoctoral research in the Department of Psychiatry and Behavioral Sciences at Emory University. Her research focused on the role of stress in the etiology of depression in animal models. Prior to joining *Nature Neuroscience*, she was an editor at *Nature Reviews Neuroscience* from 2006-2014, and a locum manuscript editor at *Nature* in 2015-2016. Leonie's research interests include psychiatric and neurodevelopmental

	disorders, sleep, innate behaviours, neural circuits and systems neuroscience.

Editorial assessment and review synthesis

**Editor's summary and assessment**	The mechanisms by which early-life adversity predisposes to eating disorders are unknown. This study focuses on LepR-expressing neurons in the lateral hypothalamus (LH), an area that modulates the hormonal stress response and plays a role in regulating food intake and motivated behaviours. The authors used 23h maternal separation to model early-life trauma (ELT), and assessed both female and male ELT and control (non-ELT) mice. Adult ELT mice show normal BW and behaviour, but when binge eating was induced (through scheduled intermittent access to HFD), they showed exaggerated and more-sustained binge-eating responses and gained more weight than non-ELT mice. The authors provide data supporting a model in which ELT => reduced LepR expression in LH => increased activity of (GABAergic) LepR+ LH neurons upon repeated HFD exposure => increased inhibition of vlPAG(Penk) neurons => increased binge-eating upon repeated HFD exposure (but not outside of that paradigm).<br><br>The editors deemed the paper to be potentially of high interest due to the combination of careful circuit manipulations, early-life stress and the focus on binge eating. However, the novelty is somewhat compromised by an earlier paper implicating a LH(GABA)=>vlPAG(GABA) projection in food-intake regulation [PMID: 31315042], which should be cited. The editors also note that chemogenetic activation of vlPAG(pENK) neurons was not performed in ELT mice to ascertain whether this can rescue the exaggerated binge-eating phenotype.<br><br>As part of the Guided Open Access pilot, editors from *Nature, Nature Neuroscience* and *Nature Communications* have discussed the reviewer reports and the manuscript's suitability for our journals. After careful evaluation, our editorial recommendation is to revise the manuscript and submit back through the Guided Open Access submission portal for consideration at *Nature Neuroscience* or *Nature Communications*.

**Editorial synthesis of reviewer reports**	The paper has been seen by three reviewers. They are positive about the advance provided by the paper, but all three reviewers raise a number of technical concerns that will need to be addressed in order to increase the robustness of the data and strengthen the conclusions.<br><br>To be considered further at *Nature Neuroscience* you would need to address all the reviewer concerns, including Reviewer #3's request to provide further insight into the interaction between ELT and activity of the identified pathway in response to repeated HFD exposure.<br><br>To be considered further at *Nature Communications* you would need to address all the reviewer concerns, although they would not strictly require you to address Reviewer #3's major point 1.

Editorial recommendation

Nature<br><br>Revision not invited	Following editorial assessment of the paper and reviewer reports it was felt that the conceptual advance is not sufficient for further consideration at *Nature*.
Nature Neuroscience<br><br>Major revisions with extension of the work	The editors would expect to see all major points addressed with additional data/analyses, including reviewer #3's request to provide some insight into the interaction between ELT and activity of the identified pathway in response to repeated HFD exposure.
Nature Communications<br><br>Major revisions	The editors find that the authors need to experimentally address most of the reviewers' comments with additional data/analyses. While they encourage you to also address Reviewer #3's major point 1, this would not be strictly necessary.

Next steps

**Editorial recommendation 1:**	Our top recommendation is to revise and resubmit your manuscript to *Nature Neuroscience.*
**Editorial recommendation 2:**	You may also choose to revise and resubmit your manuscript to *Nature Communications.* This option might be best if the requested experimental revisions are not all possible/feasible at this time.

Revision

To follow our recommendation, please upload the revised manuscript files using **the link provided in the decision letter**. Should you need assistance with our manuscript tracking system, please contact Adam Lipkin, our Nature Portfolio Guided OA support specialist, at guidedOA@nature.com.

Revision checklist

- ☐ Cover letter, stating to which journal you are submitting
- ☐ Revised manuscript
- ☐ Point-by-point response to reviews
- ☐ Updated Reporting Summary and Editorial Policy Checklist
- ☐ Supplementary materials (if applicable)

Submission elsewhere

If you choose not to follow our recommendations, you can still take the reviewer reports with you.

Option 1: Transfer to another Nature Portfolio journal
Springer Nature provides authors with the ability to transfer a manuscript within the Nature Portfolio, without the author having to upload the manuscript data again. To use this service, **please follow the transfer link provided in the decision letter.** If no link was provided, please contact guidedOA@nature.com.

> *Note that any decision to opt in to In Review at the original journal is not sent to the receiving journal on transfer. You can opt in to In Review at receiving journals that support this service by choosing to modify your manuscript on transfer.*

Option 2: Portable Peer Review option for submission to a journal outside of Nature Portfolio
If you choose to submit your revised manuscript to a journal at another publisher, we can share the reviews with another journal outside of the Nature Portfolio if requested. You will need to request that the receiving journal office contacts us at guidedOA@nature.com. We have included editorial guidance below in the reviewer reports and open research evaluation to aid in revising the manuscript for publication elsewhere.

Annotated reviewer reports

The editors have included some additional comments on specific points raised by the reviewers below, to clarify requirements for publication in the recommended journal(s). However, please note that all points should be addressed in a revision, even if an editor has not specifically commented on them.

Reviewer #1 information	
**Expertise**	Regulation of feeding and reward, animal models of eating disorders
**Editor's comments**	This reviewer is overall positive about the study, but raises concerns about the low number of animals or cells in some experiments, and is not convinced by the efficiency and specificity of the shRNA-mediated LepR knock-down.

Reviewer #1 comments	
**Section**	**Annotated Reviewer Comments**
**Remarks to the Author: Overall significance**	In this manuscript by Shin et al, a leptin sensitive LH-PAG pathway is discovered that appears to be an essential role in early life stress to increase bingeing on HFD later in life. A nice set of experiments ranging from mapping of activity, in vivo recording and opto- and chemogenetic stimulations and inhibition result in a synthesis of evidence for this projection to play a role in this bingeing.<br>The paper is well written, the data are novel and appropriately introduced and discussed.
**Remarks to the Author: Impact**	
**Remarks to the Author: Strength of the claims**	Major:<br><br>1) In Fig 1 g please show pictures of whole hypothalamus, was fos staining specific for LH in HFD? Also in extended data fig 2d show examples of each brain region.<br><br>2) There is not enough information on the AAV vector with shRNA against LepR: is there proof of efficacy and specificity? GFP is not a good control here, a shRNA control is missing. How many shRNAs were tested and how do we know that there are no off-target effects? shRNAs are known to saturate the endogenous RNAi pathway resulting in non specific effects. A CrispR/cas method or floxed LepR allele would be a convincing proof to demonstrate the importance of LepR in LH. More evidence is required here.<br>**See also referee 2 comment #3. Evidence for involvement of the LepR in LH will have to be strengthened in the revised manuscript.**<br><br>3) Extended Data Fig. 1: Measures of leptin and corticosterone levels are performed with n=3 and 4 per groups. Would it be possible to increase the sample size? At least, a non-parametric test should be performed with this very low n and not a t-test.

Please increase the sample size by including an additional 4 animals/group (see also referee 3's comment B).

The authors write : « Together, these data support the hypothesis that the LHLepr neurons of ELT mice are tirelessly activated to the repeated Re-HFD exposures, which may lead to sustained binge-like eating habits." ». Tirelessly is not in line with what is shown in fig 3 and exaggerated.

4) Did the authors study intrinsic excitability of LepR neurons since they have a reduction of LepR mRNA in the LH of ELT animals and they mentioned in the discussion: "Lepr signaling modulates voltage-dependent Kv2.1 or KATP channel activity, which plays a role in regulating intrinsic neuronal excitability" page 24. The I/E balance gives only information on neuronal inputs, not neurons themselves. However, in the methods, a measure of rheobase is mentioned. Maybe they performed these analyses, what are the results about intrinsic excitability? It remains unresolved how ELT results in hyperresponsiveness of LH neurons which is a weak point.

Please provide data indicating whether ELT increases intrinsic excitability of LepR LH neurons.

5) It seems from fig 3d and g that from the total population of cells only a few drive the response to HFD exposure. The total amount of analyzed cells is rather low. It would be better to time-lock fluorescence signal to approach to food. The analysis is not clear. Was the stats done on only those cells that responded for e and h? Was the stats done on ELT and control animals together? What was the unit of analysis (animal or cell)?

See also referee 2 comment #4. The analyses of these imaging data should be clarified and expanded to provide additional insight into the response of LepR LH neuron subtypes to repeated HFD in ELT vs control mice.

6) The authors found a remarkable and selective effect only when manipulating the LepRvlPAG pathway. The authors also found that this pathway sends collaterals to the VTA. They excluded any VTA implication based on results obtained in control animals (Fig 5-a-f and Extended Data Fig. 7.e-h). Did they test it in ELT animals? Indeed, in Fig 5 g-j, they obtained a very nice rescue with chemogenetic manipulation in ELT animals but we cannot exclude VTA implication here. In addition important controls are missing: what happens to weight gain when the LepRvlPAG pathway is hyperpolarized without ELT?

7) In Fig. 6.l-m, in a similar way as in the earlier comment, what happened when chemogenetic activation of vlPAGPenk neurons is performed in ELT animals? The figure 6 only test it in control animals and in animals under chronic HFD exposure (Fig 6l-m).

8) The authors concluded: "In conclusion, we have delineated a novel

	pathway—through LHLepr neuronal projections to the vlPAG—that is necessary and sufficient for the binge-like eating habits and HFD-induced obesity associated with ELT." Considering comments above, this should be mitigated as the association between LHLepr to vlPAG and ELT has not been fully tested (not necessary and sufficient).<br>**W.r.t. points 6-8, please provide additional evidence showing that increased activity of the LH(LepR)=>vlPAG pathway and the resulting chronic inhibition of vlPAG(pENK) neurons *in ELT mice* underlies their increased binge-eating phenotype.**<br><br>9) In the discussion the authors compare early adverse events in childhood to ELT at day 3 in mice. These developmental periods do not overlap between mice and man. The authors should comment on the limitations of this comparison<br><br>Minor:<br><br>1) Page 8 (referring to extended fig.1 k and l): 1) The legend colors do not fit the histograms colors. 2) The increase of HF food intake when animals are re-exposed to HFD for a second time (compered to the 1st exposure group) could be due to a "decrease of novelty" effect rather than a binge-like eating as mentioned in the text (page 8, line 4-5).<br><br>2) Page 8, Line 3 NC to define (normal chow), first appearance in the text<br><br>3) Fig.2h: it is mentioned in the text that the results represent a peak of evoked synaptic inputs but nothing is said about how the authors did evoke it in the material and methods (e.g. electrically, optogenetically).<br><br>4) Page 25: Could the authors discuss the potential link between the down-regulation of Leptin receptors and the enhanced LHLepr neuronal activity as shown by the increase E/I ratio they found?<br><br>5) Was normal chow always available (also when HFD was given so that it was a choice diet)<br><br>6) When did HFD start, what was the age of the mice
**Remarks to the Author: Reproducibility**	At many places n is rather low and statistical analysis not clear as indicated in comments.<br>**See also referee 3's comment B.**

Reviewer #2 information

**Expertise**	stress, reward, technologies
**Editor's comments**	This reviewer expresses a high level of enthusiasm for the study, but notes several aspects of the study can be improved, in particular regarding the analysis of the Ca2+ imaging in Figure 3 and the interpretation of the fiber density data in Figure 4.

Reviewer #2 comments

Section	Annotated Reviewer Comments
**Remarks to the Author: Overall significance**	In this manuscript by Shin et al in the BK Lim lab, the authors establish a link between early adversity and binge-like eating by showing the recruitment of a lateral hypothalamus and brainstem (PAG/LDR) circuit. This is a very beautifully conducted, thorough series of experiments, which are unique and novel yet also open several new directions for the field, alongside answering some long standing questions about the relationship between early life stress and eating disorders in general. The authors use a series of high resolution, parallel cutting-edge methods, to uncover novel insights, and further implicate opioidergic containing neurons in brainstem in these processes.
**Remarks to the Author: Impact**	The manuscript provides several new avenues for further research in the field and also links with recent efforts in the field to understanding LH circuits in feeding with those which have been recently identified in regards to state-dependent changes in feeding (i.e., Castro et al, 2021, Nature, among others).
**Remarks to the Author: Strength of the claims**	Overall the study is well conducted and conclusions are conservative, however there are some experiments and analysis, alongside discussion points that should be addressed.<br><br>Major-<br>1) It was surprising to see the results in the supplemental figure 1 that the ELT did not produce other changes in anhedonia, anxiogenesis or otherwise? The authors would be advised to also report or test whether the animals show sensitivity to stressors following ELT to demonstrate the efficacy of the model. A simple experiment to show stress-induced changes in elevated plus or open field data may suffice, but the others should provide more evidence that there was a long lasting state change following ELT which would be consistent with other literature. This should also be discussed in the discussion as well.<br>**Please provide these data to validate the ELT phenotype in terms of stress sensitivity; this may require increasing the n (see also referee**

3's comment B).

2) Were the authors surprised that with a change in Cort at P4 that there was no concomitant change in daily behavioral cycles show in EX Data F2? Wouldn't one expect that a change in cort would result in changes in both physiology and behavior in light and dark phases as has been previously reported. Some more explanation and/or evidence from the mice at an early time point may be revealing and substantiate their claim.

Please address this point through a brief discussion in the manuscript text.

3) Figure 2 is an exciting set of data, yet it would be helpful for the authors to consider also using LepR antagonists in the region to substantiate the data. Was their a particular reason they avoided using that as another control which would avoid compensatory effects in some ways? The Extended Data generally show a lack of changes in several key genes, but the list isn't exhaustive and certainly LepR being a TK receptor could have many effects on a variety of other genes and pathways. If the pharmacology experiment is too challenging the authors should address this caveat more in the discussion.

See also referee 1 comment #2.

4) The imaging data presented are very interesting, but leave the reader wondering several questions and could be more thoroughly analyzed. It would be useful to see more examples of individual neuron traces, a better sense of how reliably they can track neurons over time, and some sense of other types of responses they are seeing. The LepR-cre driver is likely to label both vGAT and vGlut+ neurons, which means there is some explanation of the up and down response they see in 3F and I. However, there are additional LepR subtypes of neurons and yet we only see mostly a binary response in the behavior. This particular figure could be filled out a bit more with more analysis and clustering approaches including more raw data so that the readers can fully appreciate the high resolution method which was used in this case.

See also referee 1 comment #5.

5) One major concern is the interpretation of the data presented in Figure 4. Fiber density quantification is always a bit misleading (at best), and only reports in reality the efficacy or "tropism" of a virus at infecting a certain "circuit". The authors should use another non-viral method to show the tracing they report is consistent if they would like to make claims about fiber density. Furthermore, if there isn't any synaptic connectivity ephys data, if we assume "more fibers = more powerful circuit" we are assuming incorrectly. Transmitters are released and amplified by receptors, and each circuit may have a variety of constraints and differences in that regard. So I would advise either providing addtional tracing data using other methods or down playing the meaning of the "fiber density". The authors should know that this reviewer agrees that this is common in the field, however, it is technically misleading nevertheless, and

	that false concept is propagated further by F3c and F3F in particular. Also the images in the VTA, DR and PAG should be brightened more to see the fibers more easily.<br><br>6) The choice of inhibitory opsin is somewhat concerning given the widely reported challenges with pumps. Cl- gradiants and rebound excitations, etc? Do the authors have any other ways of showing necessity in this circuit which might alleviate this result? A Gi DREADD experiment with local CNO for example may provide more confidence? Or perhaps a pharmacological local infusion with stimulation to establish the effect? The fDIO experiment sort of addresses this point, but those Gi-receptors can still travel elsewhere if axon collaterals are present. The authors should address and/or discuss this limitation in the manuscript.<br><br>7) The findings in figure 6, are particularly interesting especially in light of a recent report (Castro et al, 2021, Nature), whereby these same PENK neurons are shown to elevate feeding when inhibited by a Gi-coupled MOR receptor, and DREADDs as well. How confident are the authors in the anatomical localization of these neurons, they certainly look like a very similar population to those studied in a recent report, and it would be interesting to discuss the similarities of the finding with that recent data set.<br><br>Finally the floor effect of the Gq DREADD is a bit surprising and incredibly robust? Are the animals moving around in this experiment, what were some additional controls the authors considered given this very strong effect.<br><br>Minor-<br><br>- There are some typos throughout which should be corrected, mostly in the intro and discussion, just another round of proof reading will do them well.<br>- Some of the anatomy images seem dim, while when transferred to Ai can be easily enhanced or brightened. Given how printing can dim them further and press changes, I encourage the authors to brighten some of the dimmer anatomical images to enhance visibility, without changing the conclusions.
**Remarks to the Author: Reproducibility**	I had no major concerns about reproducibility or statistical matters. I think there is a limited amount of analysis on the calcium imaging data including specific details about clustering of neuronal ensembles, tracking neurons across time, and so forth. This isn't a concern with the analysis they show per se, but the authors should consider using some more advanced analysis methods to examine the individual neuronal responses more carefully. The authors should be commended on their figure presentation style, their transparency in the EX Data, and the added efforts to show hits, anatomy and full details. Many papers at this level do not do this in a careful way, and this reviewer appreciates that level of detail provided.

Reviewer #3 information	
**Expertise**	Regulation of feeding and reward
**Editor's comments**	This reviewer is also positive about the study, but raises concerns about the low n, the viral targeting data, and the absence of insight into the interaction between ELT and activity of the LH(LepR)=>vlPAG(pENK) pathway in the response to repeated HFD.

Reviewer #3 comments	
**Section**	**Annotated Reviewer Comments**
**Remarks to the Author: Overall significance**	The authors describe a leptin-receptor expressing set of neurons in the lateral hypothalamus that can modulate binge behavior (seen in early life stress models) via projections to the ventral lateral periaqueductal gray. The analysis is broad and includes studies of neural activity and neural cell type/circuit function in behavior. These are novel findings and should be of broad interest for scientists studying stress, binge eating, lateral hypothalamus and periaqueductal gray. The authors have placed the work into context in a balanced way though as noted below there is limited effort to connect the mechanistic (circuit) findings back to the early life stress.
**Remarks to the Author: Impact**	The authors describe a leptin-receptor expressing set of neurons in the lateral hypothalamus that can modulate binge behavior (seen in early life stress models) via projections to the ventral lateral periaqueductal gray. The analysis is broad and includes studies of neural activity and neural cell type/circuit function in behavior. These are novel findings and should be of broad interest for scientists studying stress, binge eating, lateral hypothalamus and periaqueductal gray. The authors have placed the work into context in a balanced way though as noted below there is limited effort to connect the mechanistic (circuit) findings back to the early life stress.
**Remarks to the Author: Strength of the claims**	Overall, this is a comprehensive effort with analysis on neuronal, circuit, intracellular, and behavioral levels. However, I do have several concerns as outlined here about interpretation and claims. While I do not think any of the experiments are required per se, the order reflects their importance and some claims would have to be changed in their absence (as noted)<br><br>1- The paper starts by presenting data showing how early life stress (or early life trauma- ELT) interacts with binge eating. Specifically, ELT increases intake in response to repeated exposure to high-fat diet (HFD). The authors then focus

	on this repeated exposure (binge) for much of the remaining manuscript. While this yields some interesting results, there do not return to the ELT and connect it to the mechanisms found here. For example, are the vlPAG(penk) neurons modified by ELT? Or does modulation of the LH-vlPAG pathway described here enhance or offset the effects of ELT? Again, I think the data on binge are valuable but the emphasis in the title and background seems to be on ELT and the work does not reconnect. While this is not essential, its absence limits impact and would require a re-writing of the introduction and abstract to reflect the emphasis of the work.<br>**This is reminiscent of referee 1 comment #6-8. Rather than modifying the title and abstract, please provide additional data to show whether/how ELT modulates the LH(LepR)=>vlPAG pathway resulting in chronic inhibition of pENK neurons in vlPAG and, thereby, increases binge-eating. These experiments would not be strictly necessary for consideration at Nature Communications.**<br><br>2- The behavioral results are generally robust but there is a potential role for novelty (upon repeated exposure) that should be considered. For example, the response of AAV-Lepr shRNA (in LH) animals to the re-HFD could be caused by a different perception of how novel the food is. That is, the AAV-Lepr animals might not be remembering the HFD. This could be tested by looking at novelty response (novel object test) or more specifically by exposing them repeatedly to a normal diet but with different flavor. This same question applies to the activity analysis of LH Lepr neurons (Fig. 3) where testing non-food items would be valuable to help with interpretation. These experiments are important for interpretation of the behavioral results.<br><br>3- Figure 5 presents data using both optogenetics and chemogenetics to manipulate the LH(lepr)-VlPAG circuit but the presentation makes it difficult to compare results to each other and to previous experiments. In particular, the eNpHR3.0 inhibition experiment uses a real time assessment of feeding which makes sense with the approach, but the results are in isolation from the other analyses in the paper. Also, the authors do not seem to look at repeated exposures for the inhibition studies. While the data here are valuable the presentation is not parallel, and it is difficult to compare inhibition and excitation of this circuit.<br><br>4- The authors state that “these c-fos data suggest that the LH or Arc activation is crucial for sustaining the binge-like eating of ELT mice.” This should be re-worded to reflect that c-fos data, as a correlative measure, is simply suggesting the potential role of the region.
**Remarks to the Author: Reproducibility**	A- The authors do not present any data on targeting for the large number of viral experiments here. It is necessary to both describe the criteria for inclusion, note how this was assessed, note the numbers removed for each

	experiment, and preferably show examples and/or summary data for the analysis.<br>**Please also clarify whether any data exclusions were determined by someone who was blind to the group assignments..**<br><br>B- The authors should increase the number of animals in some experiments. The differences seen in leptin levels in Extended Fig. 1a are not believable with n=3-4 as presented. This is also the case with some of the "negative data" that show no changes. For example, the lack of change in corticosterone in ELT animals (Ext Data Fig. 1j) or RER (Ext Data Fig. 2C) are not compelling with the number of animals analyzed. Likewise, the complex experimental designs in Figure 4(g-j) and Figure 5 contain group numbers as low as 4 raising concerns about statistical power.<br>**See also referee 1 comment #3, and referee 2 comment #1.**<br><br>C- The Cre and Lepr co-localization in Ext Data Fig 4a is not compelling. I will acknowledge that this analysis can be challenging (and many groups do not even try this) but a Cre signal, though low, appears to be present in the wildtype animals and co-localizing with Lepr<br><br>D- Extended Data Fig. 1l - the legends labels do not match the bar graph.

Open research evaluation

General information

Guidelines for Transparency and Openness Promotion (TOP) in Journal Policies and Practices ("TOP Guidelines")

The recommendations and requests in the table below are aimed at bringing your manuscript in line with common community standards as exemplified by the TOP Guidelines. While every publisher and journal will implement these guidelines differently, the recommendations below are all consistent with the policies at Nature Portfolio. In most cases, these will align with TOP Guidelines Level 2.

FAIR Principles

The goal of the recommendations in the table below related to **data or code** availability is to promote the FAIR Guiding Principles for scientific data management and stewardship (*Scientific Data* **3**: 160018, 2016). The FAIR Principles are a set of guidelines for improving 4 important aspects of digital research objects: **F**indability, **A**ccessibility, **I**nteroperability and **R**eusability.

ORCID

ORCID is a non-profit organization that provides researchers with a unique digital identifier. These identifiers can be used by editors, funding agencies, publishers, and institutions to reliably identify individuals in the same way that ISBNs and DOIs identify books and articles. Thus the risk of confusing your identity with another researcher with the same name is eliminated. **The ORCID website** provides researchers with a page where your comprehensive research activity can be stored.

Springer Nature collaborates with the ORCID organization to ensure that your research contributions (as authors and peer reviewers) are correctly attributed to you. Learn more at https://www.springernature.com/gp/researchers/orcid

**Data availability**
**Data Availability Statement**

Many journals, including all Nature Portfolio journals, require a Data Availability Statement in the manuscript as a condition of publication. The Data Availability Statement should be as detailed as possible and include accession codes or other unique IDs for deposited data, information about where source data can be found, and specify any restrictions to data access that may apply. At a minimum, the statement should indicate that data are available upon request and explain how data access can be granted. If data access is not possible, the reasons for this must be made clear in the Data Availability Statement.

More information about the Nature Portfolio data availability policy can be found here: https://www.nature.com/nature-portfolio/editorial-policies/reporting-standards#availability-of-data

Additional information about Data Availability Statements and Springer Nature's data policies are available here: http://www.springernature.com/gp/authors/research-data-policy/data-availability-statements/12330880

Other data requests

In line with community standards regarding open research, Springer Nature strongly supports data sharing and believes that all datasets on which the conclusions of the paper rely should be available to readers. We encourage authors to ensure that their datasets are either deposited in publicly available repositories (where available and appropriate) or presented in the main manuscript or additional supporting files whenever possible.

To learn more about data sharing and recommended data repositories, please see https://www.springernature.com/gp/authors/research-data-policy/repositories/12327124

Ethics

We believe that Springer Nature has a responsibility to support the relevant guidelines (based on research community or geographical region) that specify best practice in research and thus require all experimental results on animal and human participants to conform to the authors' local regulations and ethical standards, and we also encourage adherence to international standards.

Because your study uses live vertebrates, a statement affirming that you have complied with all relevant ethical regulations for animal testing and research is necessary. A statement explicitly confirming if the study received ethical approval, including the name of the board and institution that approved the study protocol is also required. The species, strain, sex and age of animals should be included.

Further details on our policies can be found at https://www.nature.com/commsbio/editorial-policies/ethics-and-biosecurity

Reporting & reproducibility

We believe that research publications should adhere to high standards of transparency and robustness in their methods and results. This, in turn, supports the principle of reproducibility, which is a foundation of good research, especially in the natural sciences. All data that support the conclusions drawn must be presented in the manuscript unless they are published elsewhere.

Nature Portfolio journals do not allow statements of “data not shown”. Please remove these statements or provide the relevant data.

Statistical reporting

Wherever statistics have been derived (e.g. error bars, box plots, statistical significance) figure legends should provide and define the n number (i.e. the sample size used to derive statistics) as a precise value (not a range), using the wording “n=X biologically independent samples/animals/cells/independent experiments/n= X cells examined over Y independent experiments” etc. as applicable. The figure legends must also indicate the statistical test used. Where appropriate, please indicate in the figure legends whether the statistical tests were one-sided or two-sided and whether adjustments were made for multiple comparisons. For null hypothesis testing, please indicate the test statistic (e.g. F, t, r) with confidence intervals, effect sizes, degrees of freedom and P values noted.

All error bars need to be defined in the figure legends (e.g. SD, SEM) together with a measure of centre (e.g. mean, median). For example, the legends should state something along the lines of “Data are presented as mean values +/- SEM” as appropriate. All box plots need to be defined in the legends in terms of minima, maxima, centre, bounds of box and whiskers and percentile.

For examples of expected description of statistics in figure legends, please see the following:
https://www.nature.com/articles/s41467-019-11636-5
or
https://www.nature.com/articles/s41467-019-11510-4

When describing results as "significant" in the main text, please include details about the statistical test used and provide an exact p-value, rather than a significance threshold.

We strongly discourage deriving statistics from technical replicates, unless there is a clear scientific justification for why providing this information is important. Conflating technical and biological variability, e.g., by pooling technically replicates samples across independent experiments is strongly discouraged.

Data presentation

The quality of some of the figures appears to be quite low. If possible, we suggest replacing these with higher-resolution images.

	EDITORIAL REQUESTS:	AUTHOR RESPONSE:
**1.**	**Data presentation:** Please ensure that data presented in a plot, chart or other visual representation format shows data distribution clearly (e.g. dot plots, box-and-whisker plots). When using bar charts, please overlay the corresponding data points (as dot plots) whenever possible and always for n ≤ 10. (Please see the following editorial for the rationale behind this request and an example https://www.nature.com/articles/s41551-017-0079).	
	**Panels requiring revision:**<br><br>Please note that data presentation has to be revised to comply with our policy in figures 4c, f.	**Author response:**
**2.**	**Statistics:** Wherever statistics have been derived (e.g. error bars, box plots, statistical significance) the legend needs to provide and define the n number (i.e. the sample size used to derive statistics) as a precise value (not a range), using the wording “n=X biologically independent samples/animals/cells/independent experiments/n= X cells examined over Y independent experiments” etc. as applicable.	
	**Legends requiring revision:**<br><br>1. Please note that this information is missing in the legends of figures 4c, f.	**Author response:**
**3.**	Statistics such as error bars, significance and p values cannot be derived from n<3 and must be removed from all such cases.	
	We strongly discourage deriving statistics from technical replicates, unless there is a clear scientific justification for why providing this information is important. Conflating technical and biological variability, e.g., by pooling technically replicates samples across independent experiments is strongly discouraged. (For examples of expected description of statistics in figure legends, please see the following https://www.nature.com/articles/s41467-019-11636-5 or https://www.nature.com/articles/s41467-019-11510-4).	

<table>
<tr><td></td><td colspan="2">All error bars need to be defined in the legends (e.g. SD, SEM) together with a measure of centre (e.g. mean, median). For example, the legends should state something along the lines of “Data are presented as mean values +/- SEM” as appropriate.
All box plots need to be defined in the legends in terms of minima, maxima, centre, bounds of box and whiskers and percentile.</td></tr>
<tr><td></td><td><u>Legends requiring revision:</u>

1. Please note that the box plots need to be defined in terms of minima, maxima, bounds of box and whiskers in the legends of figures 5c-f; 6j; extended data figures 7a-d.

2. Please note that the box plots need to be defined in terms of minima, maxima, centre, bounds of box and whiskers and percentile in the legends of extended data figures 7f-h.</td><td><u>Author response:</u></td></tr>
<tr><td>4.</td><td colspan="2">The figure legends must indicate the statistical test used. Where appropriate, please indicate in the figure legends whether the statistical tests were one-sided or two-sided and whether adjustments were made for multiple comparisons.
For null hypothesis testing, please indicate the test statistic (e.g. F, t, r) with confidence intervals, effect sizes, degrees of freedom and P values noted.
Please provide the test results (e.g. P values) as exact values whenever possible and with confidence intervals noted.</td></tr>
<tr><td></td><td><u>Legends requiring revision:</u>

1. Please indicate the statistical test used for data analysis and where appropriate, please specify whether it was one-sided or two-sided and whether adjustments were made for multiple comparisons, in the legends of figure 4j; extended data figures 1f-j; 2a-c; 6s, t; 7a-d, f-h; 8h-l.

2. Please note that the exact p value should be provided, when possible, in the legends of figures 1d, f; 2b, i; 3e, h; 4h; 5j; 6l, m; extended data figures 1l; 2d; 3c, e, q; 8n.

3. Please indicate what ‘****’ represents; if this represents p value, please indicate the statistical test used and where appropriate, specify whether it was one-sided or two-sided and whether adjustments were made for multiple comparisons and the exact p value in the legend of figure 1h.

4. Please note that for the figures 5e, f, p-values and statistical tests are indicated in the legends. However, comparison for the same, has not been represented in the figures. Please rectify this in the figures or legends as applicable.</td><td><u>Author response:</u></td></tr>
</table>

<table>
<tr><td rowspan="2">5.</td><td colspan="2"><u>Reproducibility:</u> Please state in the legends how many times each experiment was repeated independently with similar results. This is needed for all experiments, but is particularly important wherever results from representative experiments (such as micrographs) are shown. If space in the legends is limiting, this information can be included in a section titled “Statistics and Reproducibility” in the methods section.</td></tr>
<tr><td><u>Legends requiring revision:</u>

Please note that this information is missing in the legends of figures 2c; 3a; 5a, g, i; 6c-i, k; extended data figures 3g, o; 4a, c-e; 6b-f, h-l; 8a, d-f.</td><td><u>Author response:</u></td></tr>
<tr><td rowspan="2">6.</td><td colspan="2"><u>Data availability:</u> This journal strongly supports public availability of data and custom code associated with the paper in a persistent repository where they can be freely and enduringly accessed or as a supplementary data file when no appropriate repository is available. If data and code can only be shared on request, please explain why in your data Availability Statement, and also in the correspondence with your editor. For more information, please refer to https://www.nature.com/nature-research/editorial-policies/reporting-standards#availability-of-data</td></tr>
<tr><td>Please ensure that datasets deposited in public repositories are now publicly accessible, and that accession codes or DOI are provided in the "Data Availability" section. As long as these datasets are not public, we cannot proceed with the acceptance of your paper. For data that have been obtained from publicly available sources, please provide a URL and the specific data product name in the data availability statement. Data with a DOI should be further cited in the methods reference section.</td><td><u>Author response:</u></td></tr>
<tr><td>7.</td><td colspan="2"><u>Micrographs</u>: Please ensure that all micrographs include a scale bar and this scale bar is defined on the panels or in the figure legends.</td></tr>
</table>
