# Supplementary material for: Early adversity promotes binge-like eating habits by remodeling a leptin-responsive lateral hypothalamus–brainstem pathway
Source: Nat Neurosci. 2022 Dec 12;26(1):79–91. doi: 10.1038/s41593-022-01208-0 (PMC9829538; doi:10.1038/s41593-022-01208-0)
Supplement: Supplementary file 2 — Reporting Summary [file 41593_2022_1208_MOESM2_ESM.pdf]

nature portfolio

Corresponding author(s): Byungkook Lim, Sora Shin

Last updated by author(s): Oct 12, 2022

Reporting Summary

Nature Portfolio wishes to improve the reproducibility of the work that we publish. This form provides structure for consistency and transparency in reporting. For further information on Nature Portfolio policies, see our Editorial Policies and the Editorial Policy Checklist.

Statistics

For all statistical analyses, confirm that the following items are present in the figure legend, table legend, main text, or Methods section.

n/a	Confirmed	
☐	☒	The exact sample size (*n*) for each experimental group/condition, given as a discrete number and unit of measurement
☐	☒	A statement on whether measurements were taken from distinct samples or whether the same sample was measured repeatedly
☐	☒	The statistical test(s) used AND whether they are one- or two-sided<br>*Only common tests should be described solely by name; describe more complex techniques in the Methods section.*
☐	☒	A description of all covariates tested
☐	☒	A description of any assumptions or corrections, such as tests of normality and adjustment for multiple comparisons
☐	☒	A full description of the statistical parameters including central tendency (e.g. means) or other basic estimates (e.g. regression coefficient) AND variation (e.g. standard deviation) or associated estimates of uncertainty (e.g. confidence intervals)
☐	☒	For null hypothesis testing, the test statistic (e.g. *F*, *t*, *r*) with confidence intervals, effect sizes, degrees of freedom and *P* value noted<br>*Give P values as exact values whenever suitable.*
☒	☐	For Bayesian analysis, information on the choice of priors and Markov chain Monte Carlo settings
☒	☐	For hierarchical and complex designs, identification of the appropriate level for tests and full reporting of outcomes
☒	☐	Estimates of effect sizes (e.g. Cohen's *d*, Pearson's *r*), indicating how they were calculated

Our web collection on statistics for biologists contains articles on many of the points above.

Software and code

Policy information about availability of computer code

Data collection	Doric Neuroscience Studio software (version 5.3.1.2; Doric Lenses), QuantStudio Real-Time PCR Software v1.3, pClamp 10.3, Clampfit 10.3
Data analysis	Doric Neuroscience Studio software (version 5.3.1.2; Doric Lenses), MATLAB R2015a (Mathworks), FIJI (v2.5, ImageJ, NIH), Viewer III (BIOBSERVE), ANY-maze (Stoelting Co), GraphPad Prism 6 (for stat and graph visualization), Illustrator CS4 (for generating figures), Clampfit 10.3

For manuscripts utilizing custom algorithms or software that are central to the research but not yet described in published literature, software must be made available to editors and reviewers. We strongly encourage code deposition in a community repository (e.g. GitHub). See the Nature Portfolio guidelines for submitting code & software for further information.

Data

Policy information about availability of data

All manuscripts must include a data availability statement. This statement should provide the following information, where applicable:

- Accession codes, unique identifiers, or web links for publicly available datasets
- A description of any restrictions on data availability
- For clinical datasets or third party data, please ensure that the statement adheres to our policy

Because of the size and the complexity of the data sets, the data that support the findings of this study are available from the corresponding author upon reasonable request.

Human research participants

Policy information about studies involving human research participants and Sex and Gender in Research.

Reporting on sex and gender	*Use the terms sex (biological attribute) and gender (shaped by social and cultural circumstances) carefully in order to avoid confusing both terms. Indicate if findings apply to only one sex or gender; describe whether sex and gender were considered in study design whether sex and/or gender was determined based on self-reporting or assigned and methods used. Provide in the source data disaggregated sex and gender data where this information has been collected, and consent has been obtained for sharing of individual-level data; provide overall numbers in this Reporting Summary. Please state if this information has not been collected. Report sex- and gender-based analyses where performed, justify reasons for lack of sex- and gender-based analysis.*
Population characteristics	*Describe the covariate-relevant population characteristics of the human research participants (e.g. age, genotypic information, past and current diagnosis and treatment categories). If you filled out the behavioural & social sciences study design questions and have nothing to add here, write "See above."*
Recruitment	*Describe how participants were recruited. Outline any potential self-selection bias or other biases that may be present and how these are likely to impact results.*
Ethics oversight	*Identify the organization(s) that approved the study protocol.*

Note that full information on the approval of the study protocol must also be provided in the manuscript.

Field-specific reporting

Please select the one below that is the best fit for your research. If you are not sure, read the appropriate sections before making your selection.

☒ Life sciences ☐ Behavioural & social sciences ☐ Ecological, evolutionary & environmental sciences

For a reference copy of the document with all sections, see nature.com/documents/nr-reporting-summary-flat.pdf

Life sciences study design

All studies must disclose on these points even when the disclosure is negative.

Sample size	Samples size for each experiment is described in the figure legend. Initially, sample sizes required for this study were estimated based on pilot studies or previous work (e.g., Shin et al., 2018; Neuron), but no formal statistical tests were used to predetermine sample size. However, power analysis was conducted to validate the sample size and the endpoint, according to Sample Size Determination (significance level at 0.05 and power at 0.9) from the NIH “Guidelines for the Care and Use of Mammals in Neuroscience and Behavioral Research”.
Data exclusions	If the viral expression was found outside this reference area or the viral transduction was weak in the LH (covering less than 50% of the total LH area), we excluded the mice from the final dataset, which was determined by two experimenters who were blinded to the experimental design. This exclusion happens in two wild-type mice in Extended Data Fig. 3n due to the viral expression in VMH; one wild-type mice in Extended Data Fig. 4a due to off-target cannula implantation; one Lepr-Cre mouse in Fig. 5j due to weak viral transduction.
Replication	Results were replicated in multiple trials within each animal and/or across different animals within each data set. Reproduction of the data was considered successful if the same trends were observed in the multiple trials. Experiments were replicated several rounds until we meet the criteria of power analysis.
Randomization	We need to use a group of animals with specific ages and the history of stress. However, within a group, we randomly chose animals for experiments. Animals used in this study were not selected based on any other prerequisite features other than general animal wellbeing (e.g. normal grooming and social behavior, no obvious infections, etc.) for allocation into a particular experimental group.
Blinding	Experimenters are blind to the group allocation and outcome assessment. For data analysis, primary experimenters were not blind due to the fact that the experimental conditions (e.g., stress paradigm, food exposure) were obvious to the researchers, but the analysis was carried out without the subjective bias. The video analysis in Fig 5c-f; Extended Data Fig. 6, 8a-c, 8f-i was performed by two experimenters who were blinded to the experimental design.

Reporting for specific materials, systems and methods

We require information from authors about some types of materials, experimental systems and methods used in many studies. Here, indicate whether each material, system or method listed is relevant to your study. If you are not sure if a list item applies to your research, read the appropriate section before selecting a response.

March 2021

Materials & experimental systems

n/a	Involved in the study
	☒ Antibodies
☒	Eukaryotic cell lines
☒	Palaeontology and archaeology
	☒ Animals and other organisms
☒	Clinical data
☒	Dual use research of concern

Methods

n/a	Involved in the study
☒	ChIP-seq
☒	Flow cytometry
☒	MRI-based neuroimaging

Antibodies

Antibodies used

anti-c-fos (Cell Signaling Technology; Cat# 2250S), anti-Mch (Phoenix Pharmaceuticals INC; Cat# H-070-47), ant-Hcrt (Phoenix Pharmaceuticals INC; Cat# H-003-30), anti-phospho STAT3 (Cell Signaling Technology; Cat# 9131S), Goat anti-Rabbit IgG (H+L) Highly Cross-Adsorbed Secondary Antibody, Alexa Fluor Plus 488 (Thermo Fisher Scientific; Cat# A32731), Horseradish peroxidase (HRP)-conjugated anti-rabbit secondary antibody (Cell signaling Technology; Cat# 7074S)

Validation

Anti-c-fos (Cell Signaling Technology; Cat# 2250S, RRID:AB_2247211): Validated in previous studies with many citations. Information can be found in this website. https://www.cellsignal.com/products/primary-antibodies/c-fos-9f6-rabbit-mab/2250

Anti-Mch (Phoenix Pharmaceuticals INC; Cat# H-070-47, RRID:AB_2722682): Validated in previous studies with many citations. Information can be found in this website. https://www.phoenixpeptide.com/products/view/Antibodies/H-070-47

Ant-Hcrt (Phoenix Pharmaceuticals INC; Cat# H-003-30, RRID:AB_2315019): Validated in previous studies with many citations. Information can be found in this website. https://www.phoenixpeptide.com/products/view/Antibodies/H-003-30

Anti-phospho STAT3 (Cell Signaling Technology; Cat# 9131, RRID:AB_331586): Validated in previous studies with many citations. Information can be found in this website. https://www.cellsignal.com/products/primary-antibodies/phospho-stat3-tyr705-antibody/9131

Goat anti-Rabbit IgG (H+L) Highly Cross-Adsorbed Secondary Antibody, Alexa Fluor Plus 488 (Thermo Fisher Scientific; Cat# A32731, RRID:AB_2633280): Validated in previous studies with many citations. Information can be found in this website. https://www.thermofisher.com/antibody/product/Goat-anti-Rabbit-IgG-H-L-Highly-Cross-Adsorbed-Secondary-Antibody-Polyclonal/A32731

Horseradish peroxidase (HRP)-conjugated anti-rabbit secondary antibody (Cell signaling Technology, Cat# 7074S, RRID:AB_2099233): Validated in previous studies with many citations. Information can be found in this website. https://www.cellsignal.com/products/secondary-antibodies/anti-rabbit-igg-hrp-linked-antibody/7074?site-search-type=Products&N=4294956287&Ntt=horseradish+peroxidase+%28hrp%29-conjugated+anti-rabbit+secondary+antibody&fromPage=plp

Animals and other research organisms

Policy information about studies involving animals; ARRIVE guidelines recommended for reporting animal research, and Sex and Gender in Research

Laboratory animals

C57BL6/J mice, Lepr-Cre mice (Stock No. 008320), Ai14 (Stock No. 007908; tdTom reporter line) and Penk-Cre (Stock No. 025112) mice from Jackson Laboratories. All transgenic mice for behavioral experiments were backcrossed to wild-type C57BL/6J mice for multiple generations. Both male and female mice (10-13 weeks old) were used for all experiments

Wild animals

No wild animal was used.

Reporting on sex

Both male and female animals were used.

Field-collected samples

No field collected samples were used in the study.

Ethics oversight

All experiments were carried out in accordance with the NIH guidelines and approved by the UCSD institutional animal care and use committee (IACUC) and VT institutional animal care and use committee (IACUC).

Note that full information on the approval of the study protocol must also be provided in the manuscript.

March 2021
